# Reducing daily salt intake in China by 1 g could prevent almost 9 million cardiovascular events by 2030: a modelling study

**DOI:** 10.1136/bmjnph-2021-000408

**Published:** 2022-08-16

**Authors:** Monique Tan, Feng He, Joan K Morris, Graham MacGregor

**Affiliations:** 1 Wolfson Institute of Population Health, Barts and The London School of Medicine & Dentistry, Queen Mary University of London, London, UK; 2 St George's University of London, London, UK

**Keywords:** blood pressure lowering

## Abstract

**Introduction:**

In China, salt intake is among the highest in the world (~11 g/day) and cardiovascular disease (CVD) accounts for 40% of deaths. We estimated the potential impact of reducing salt intake on CVD events in China, via systolic blood pressure (SBP).

**Methods:**

To develop our model, we extracted the effect of salt reduction on SBP from a meta-regression of randomised trials and a population study, and that of SBP on CVD risk from pooled cohort studies.

**Results:**

Reducing population salt intake in China by 1 g/day could lower the risk for ischaemic heart disease by about 4% (95% uncertainty interval 1.8%–7.7%) and the risk for stroke by about 6% (2.4%–9.3%). Should this reduced salt level be sustained until 2030,~9 million (M) (7M–10.8M) CVD events could be prevented, of which ~4M (3.1M–4.9M) would have been fatal. Greater and gradual salt intake reductions, to achieve WHO’s target of 30% reduction by 2025 or the Chinese government’s target of ≤5 g/day by 2030, could prevent ~1.5 or 2 times more CVD events and deaths, respectively. Should the prolonged effect of salt reduction over several years be accounted for, all estimates of CVD events and deaths prevented would be 25% greater on average.

**Conclusion:**

Bringing down the high salt intake levels in China could result in large reductions in CVD. An easily achievable reduction of 1 g/day could prevent ~9M CVD events by 2030. Urgent action must be taken to reduce salt intake in China.

**WHAT IS ALREADY KNOWN ON THIS TOPIC**

- Salt intake in China has consistently been high and latest figures from 24-hour urinary sodium excretion indicate it averages 11 g/day, making it one of the highest in the world and over twice the recommended maximum intake of 5 g/day by the WHO and the Chinese government. Via its effect on blood pressure, excess salt intake is a major risk factor of cardiovascular disease (CVD), which account for 40% of the deaths in China. Previous estimations of the health impact of reducing salt intake in China used either obsolete or otherwise unreliable data sources and did not account for the more prolonged effect of salt reduction on blood pressure over several years.

**WHAT THIS STUDY ADDS**

- Reducing salt intake in China, even by a modest amount of just 1 g/day, might prevent some 9 million (M) CVD events (of which almost 4M fatal) if sustained until 2030. Accounting for the more prolonged effect of salt reduction on blood pressure could result in 10M CVD events (of which more than 4M fatal) prevented.

**HOW THIS STUDY MIGHT AFFECT RESEARCH, PRACTICE AND/OR POLICY**

- A salt reduction programme that is workable, coherent, sustainable and targeting current and upcoming major dietary sources of salt in China is urgently needed. As the most populous country in the world with a population of 1.4 billion, reducing salt intake in China would also considerably improve global health.

## Introduction

Excess salt intake raises blood pressure and thus increases the risk of cardiovascular disease (CVD), which is the leading cause of death and disability in the world.^1^ The WHO has set an interim global target of reducing population salt intake by 30% by 2025 and recommends all adults reduce their salt intake to less than 5 g/day.^2^

In China, salt intake has consistently been very high^3^ and CVD accounts for 40% of all deaths.^4^ Despite various governmental campaigns since 2007,^5^ the latest estimates show that salt intake in adults still averages at 11 g/day,^6^ making it one of the highest intake levels in the world.^7^ Importantly, the slow progress made so far in salt reduction could be offset by the rapid increase in the consumption of processed and out-of-home foods that comes with urbanisation.^8^

Estimating the health gains that could be obtained by reducing salt intake would provide further evidence for the development of an effective salt reduction programme in China. The aim of this study is to estimate the potential impact of reducing salt intake on stroke and ischaemic heart disease events (IHD) in China, using the latest and most robust data sources available.

## Methods

### Salt reductions

**Figure 1:**
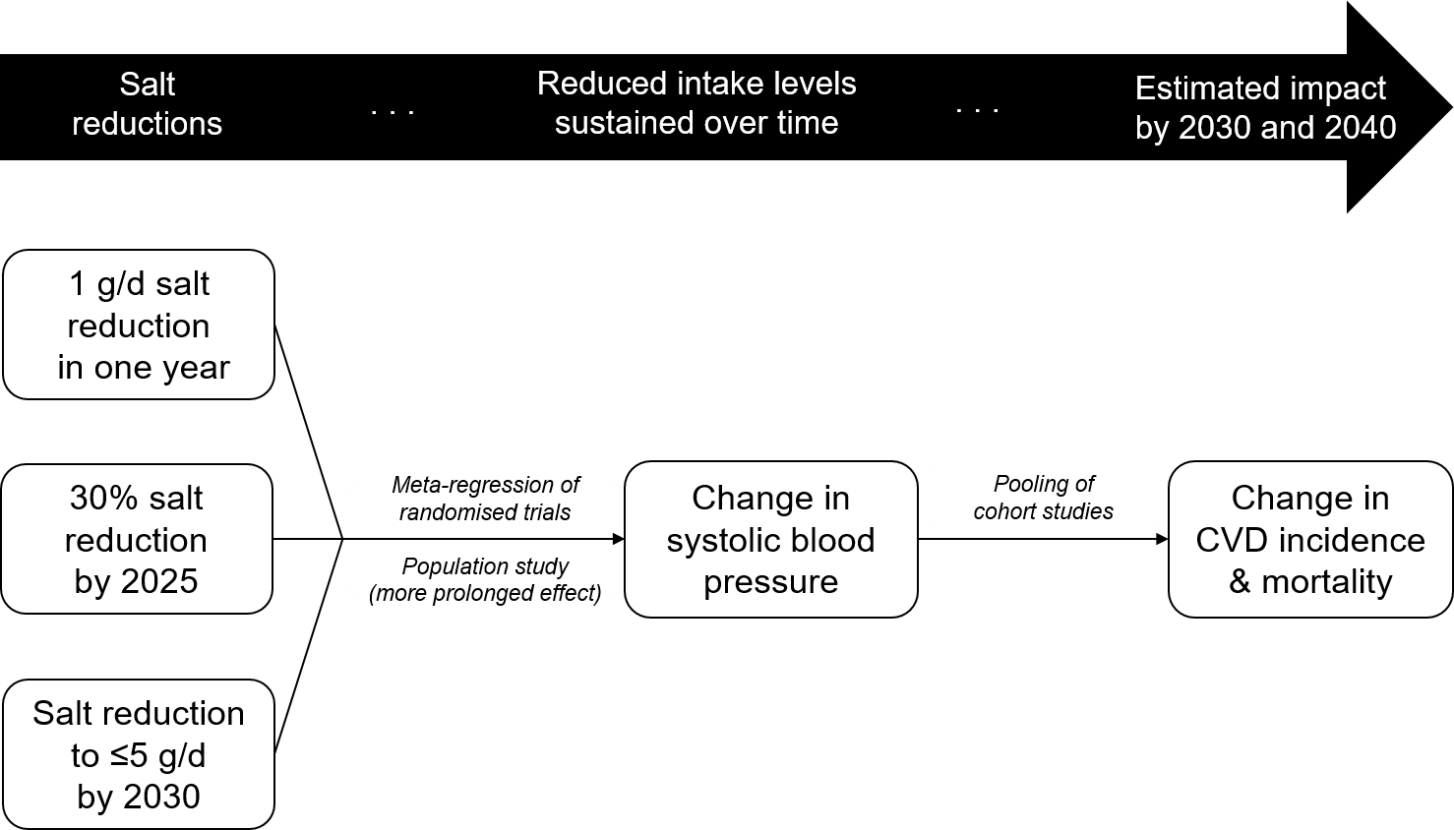
Outline of the comparative risk assessment model used to estimate the potential health impact on CVD of reducing salt intake in China. CVD, cardiovascular disease. The text in italic indicates the type of data sources used to quantify the relationships between salt intake and systolic blood pressure, and between systolic blood pressure and CVD risk.

We developed a simple comparative risk assessment model to estimate the potential health impact of reducing salt intake in China (figure 1). While excess salt consumption has been linked to an increased risk for a multitude of non-cardiovascular conditions such as gastric cancer,^1^ reliable estimates of these associations are still unavailable. Therefore, we focused on IHD and stroke, the main CVD, and refer to both IHD and stroke from now on as CVD—an approach that has been widely adopted in salt reduction modelling so far.^9–12^ There is strong and consistent evidence for a causal and dose–response relationship between salt intake and blood pressure, which is a leading risk factor for CVD. Reducing salt intake lowers blood pressure, and thus CVD risk as well.^1^

Baseline data for population size, salt intake (24-hour urinary sodium excretion), blood pressure and disease rates were taken from the latest and most robust figures available (online supplemental table 1).^6 13–20^ The data sources and their integration in the model are described in detail in the online supplemental materials.

10.1136/bmjnph-2021-000408.supp1 Supplementary data

We modelled three gradual salt reductions for the adult population of China. First, a 1 g/day reduction to be achieved in 1 year, the feasibility of which has previously been demonstrated in local salt reduction projects in parts of China.^21 22^ Second, achieving WHO’s interim target of 30% salt reduction by 2025, which is equivalent to a gradual reduction of 3.2 g/day (95% uncertainty interval (UI) 2.3–4.2) by 2025.^2^ Third, reducing salt intake to ≤5 g/day by 2030, which is the target set by the Chinese Government in its action plan for health and development, ‘Healthy China 2030’.^23^ The salt reductions to the WHO and the Chinese government targets were modelled as gradual percentage salt reductions, for example, a gradual reduction of 7% per year in salt intake to achieve a total of 30% reduction by 2025. We then estimated the falls in systolic blood pressure (SBP) levels and CVD risk, events and deaths resulting from the salt reductions, by comparing them to the levels at which they would have been without salt reduction (online supplemental figure 2).

### Health impact modelling

A two-step process was used to estimate the impact of salt reduction on CVD, following the well-established causal relationships between salt intake and SBP, and between SBP and CVD risk.^1^ First, changes in SBP following salt reduction were estimated. Second, changes in the incidence of first-ever CVD events (fatal and non-fatal, with premature deaths defined as occurring before the age of 70 years) as a result of the changes in SBP were estimated using relative risks, and applied to baseline CVD rates in the population (table 1). We derived the parameters to describe these relationships from a meta-regression of randomised trials (to quantify the effect of salt reduction on SBP in hypertensive and normotensive individuals), a population study (to quantify the more prolonged effect of salt reduction on SBP) and a pooling of cohort studies (to quantify the effect of SBP reduction on CVD risk reduction).^13 19 20^

**Table 1:** Model input parameters and data sources

	Parameter	Data source
Effect of salt reduction on systolic blood pressure:		
Based on randomised trials	Decrease in systolic blood pressure per 1 g/day salt reduction: Normotensive adults: 0.75 mm Hg (95% CI 0.20 to 1.30) Hypertensive adults: 1.89 mm Hg (95% CI 0.84 to 2.93)	Meta-regression of randomised trials of salt reduction, adjusted for age and ethnic group.^13^ Median trial duration: 4–5 weeks
Based on a population study	Decrease in systolic blood pressure per 1 g/day salt reduction: Normotensive and hypertensive adults: 1.93 mm Hg (95% CI 1.45 to 2.40)	Population study of salt reduction.^19^ Timespan observed: 8 years
Effect of systolic blood pressure change on cardiovascular disease risk:		
Ischaemic heart disease	Reduction in relative risk of first ischaemic heart disease event (fatal or non-fatal) for each 1 mm Hg systolic blood pressure reduction: 35–44 yeas: 4.0% (95% CI 2.2% to 5.5%) 45–54 years: 3.6% (95% CI 2.2% to 4.7%) 55–64 years: 3.1% (95% CI 2.2% to 3.8%) 65–74 years: 2.5% (95% CI 2.2% to 2.8%) 75–84 years: 2.1% (94% CI 1.9% to 2.2%)	Pooling of meta-analysis of epidemiological studies (Asia Pacific Cohort Studies Collaboration and the Prospective Studies Collaboration), representing a total of 1.38M participants with 65 000 cardiovascular disease events from 99 cohorts.^20^
Stroke	Reduction in relative risk of first stroke event (fatal or non-fatal) for each 1 mm Hg systolic blood pressure reduction: 35–44 years: 5.1% (95% CI 4.7% to 5.5%) 45–54 years: 4.5% (95% CI 4.2% to 4.8%) 55–64 years: 3.9% (95% CI 3.6% to 4.1%) 65–74 years: 3.1% (95% CI 2.8% to 3.3%) 75–84 years: 2.2% (95% CI 2.0% to 2.5%)	

Region-specific, age-specific and sex-specific estimates of baseline salt intake, SBP and disease rates (as shown in online supplemental table 3) were used, and subgroup-specific results are reported in the online supplemental materials. Uncertainty intervals reflect the uncertainty in baseline salt intake and SBP, disease rates, and relationships between salt intake, SBP and CVD risk. We estimated them using 5000 iterations of a Monte Carlo analysis based on the published uncertainty of each parameter estimate (online supplemental table 2). All simulations were written and performed on R V.3.5.1, using Queen Mary’s Apocrita HPC facility, supported by QMUL Research-IT. http://doi.org/10.5281/zenodo.438045.

## Results

On average, the adult population of China consumes 11.1±1.6 g/day of salt and has an SBP of 128.1±13 mm Hg.

**Figure 2:**
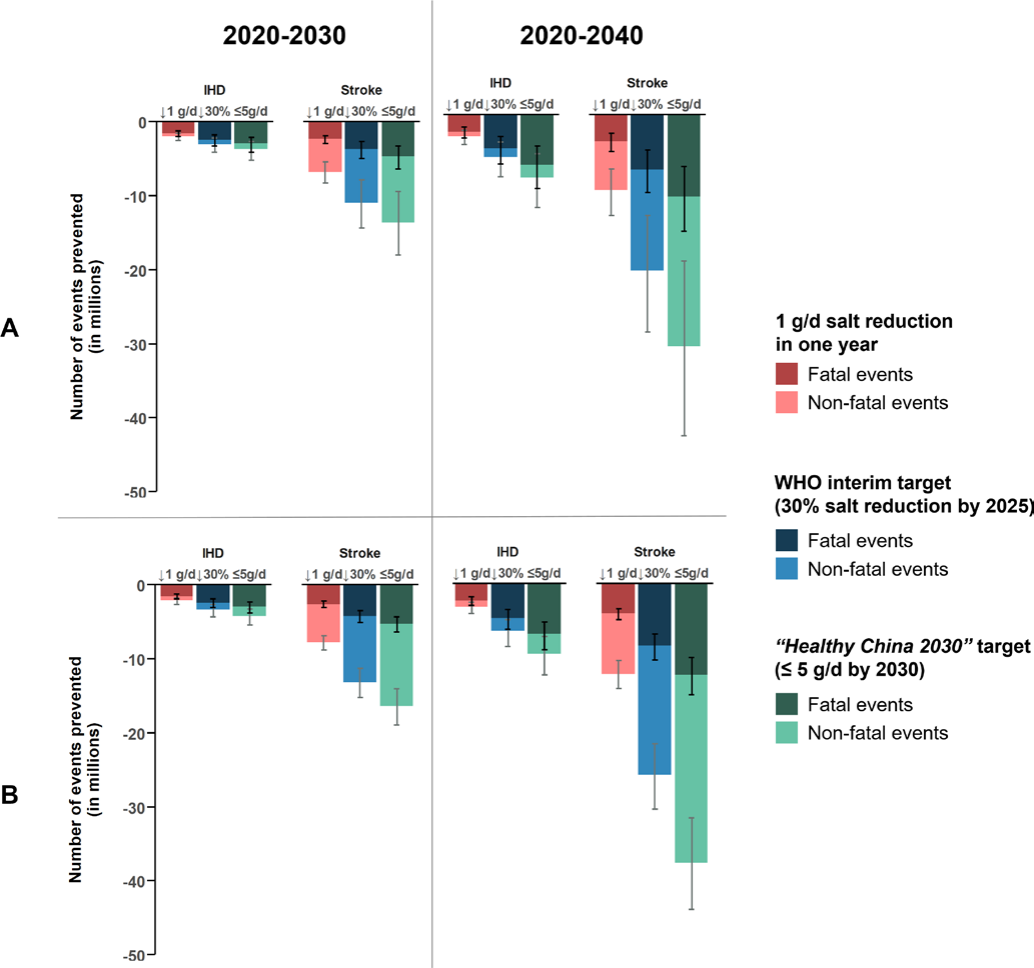
Number of CVD events and deaths prevented with different salt reductions, estimating the effect of salt reduction on blood pressure from (A) randomised trials and (B) a UK-based population study. CVD, cardiovascular disease; IHD, ischaemic heart disease.

Reducing this salt intake by 1 g/day should lower average SBP levels by approximately 1.2 mm Hg (95% UI 0.5–2.2), which could lower the risk for IHD by about 4% (95% UI 1.8–7.7) and the risk for stroke by about 6% (95% UI 2.4–9.3) (table 2). If this 1 g/day reduction was achieved in a year and the reduced salt intake levels were sustained thereafter, some 9M (95% UI 7M–10.8M) CVD events could be prevented by 2030, of which approximately 4M (95% UI 3.1M–4.9M) could have been fatal. Of those CVD deaths, about 2M (95% UI 1.6M–2.7M) could have been premature. Should the reduced salt intake levels be sustained for 10 more years, the cumulative benefits by 2040 of reducing salt intake could reach a total of approximately 13M (95% UI 9.5M–17.6M) CVD events prevented. Of these CVD events, about 6M (95% UI 4.3M–8.1M) could have been fatal, of which 3M (95% UI 2.2M–4.2M) prematurely so (figure 2, online supplemental table 4.1).

**Table 2:** Estimated change in average salt intake, SBP and cardiovascular disease risk with salt reduction

Salt reductions	Effect of salt reduction on SBP based on randomised trials				Effect of salt reduction on SBP based on a population study			
	Salt intake (g/day)	SBP (mm Hg)	IHD risk (%)	Stroke risk (%)	Salt intake (g/day)	SBP (mm Hg)	IHD risk (%)	Stroke risk (%)
1 g/day reduction in 1 year	−1 (−1 to −1)	−1.2 (−0.5 to −2.2)	−4.2 (−1.8 to −7.7)	−5.7 (−2.4 to −9.3)	−1 (−1 to −1)	−1.9 (−1.5 to −2.3)	−7.1 (−4.6 to −14)	−9.9 (−6 to −15)
30% reduction by 2025	−3.2 (−2.3 to −4.2)	−3.8 (−1.5 to −7.5)	−12.9 (−5.2 to −25.7)	−17.3 (−6.1 to −30.5)	−3.3 (−2.4 to −4.2)	−6.3 (−4.5 to −8.8)	−22 (−12.1 to −41.3)	−29.7 (−15.3 to −45.1)
Reduction to ≤5 g/day by 2030	−6 (−4.3 to −8)	−7.1 (−2.9 to −14.1)	−23 (−9.6 to −42.9)	−30.1 (−11.2 to −49.7)	−6.2 (−4.6 to −8)	−12 (−8.5 to −16.6)	−37.4 (−21.6 to −63.5)	−48.6 (−27 to −67.8)

Data are median (95% UI). The changes reported for salt intake and SBP are absolute reductions; those reported for disease risk are relative reductions.

IHD, ischaemic heart disease; SBP, systolic blood pressure.

Achieving WHO's interim target of 30% salt reduction by 2025 would require a 3.2 g/day (95% UI 2.3–4.2) total reduction in salt intake. Once achieved in 2025, average SBP levels should have fallen by approximately 3.8 mm Hg (95% UI 1.5–7.5), lowering the risk for IHD by about 13% (95% UI 5.2–25.7) and the risk for stroke by about 17% (95% UI 6.1–30.5) (table 2). Should the 2025 salt intake levels be maintained until 2030, a cumulative total of about 14M (95% UI 10M–18.5M) CVD events could be prevented by 2030, of which some 6M (95% UI 4.4M–8.3M) could have been fatal. Of these CVD deaths, approximately 3M (95% UI 2.4M–4.6M) could have been premature. Should the 2025 salt intake levels be further maintained until 2040, the cumulative total number of CVD events prevented could reach approximately 27M (95% UI 17.2M–37.8M). Of these CVD events, about 12M (95% UI 7.6M–17.2M) could have been fatal, of which some 6M (95% UI 4M–9.2M) prematurely so (figure 2, online supplemental table 4.1).

Achieving the ‘Healthy China 2030’ target of ≤5 g/day by 2030 would require an 8.2% reduction in salt intake per year. This would represent a total of 6 g/day (95% UI 4.3–8) reduction in salt intake. Once achieved in 2030, average SBP levels should have fallen by approximately 7.1 mm Hg (95% UI 2.9–14.1), risk for IHD could be about 23% (95% UI 9.6–42.9) lower, risk for stroke could be about 30% (95% UI 11.2–49.7) lower (table 2) and some 17M (95% UI 12.1M–23.2M) CVD events could have been prevented, of which some 8M (95% UI 5.3M–10.4M) could have been fatal. Of these CVD deaths, about 4M (95% UI 2.8M–5.8M) could have been premature. Should salt intake levels remain ≤5 g/day until 2040, a cumulative total of about 40M (95% UI 25.1M–55.9M) CVD events could be prevented, of which some 18M (95% UI 11.2M–25.7M) could have been fatal. Of these CVD deaths, approximately 9M (95% UI 5.8M–13.5M) could have been premature (figure 2, online supplemental table 4.1).

When we accounted for the more prolonged effect of salt reduction on SBP, the estimated health impact of salt reduction was greater (table 2, figure 2). For example, the estimated number of CVD events prevented by 2030 was approximately 10M (95% UI 8.6M–11.4M) with a 1 g/day reduction achieved in a year, 17M (95% UI 13.9M–19.5M) with a 30% salt reduction by 2025 and 21M (95% UI 17.3M–24.3M) with a reduction to ≤5 g/day by 2030. The corresponding estimates for CVD deaths prevented by 2030 were approximately 4M (95% UI 3.4M–4.9M), 7M (95% UI 5.4M–8.2M) and 8M (95% UI 6.7M–10.2M) (online supplemental table 4.2).

All region, age and sex subgroups would benefit from reducing their salt intake (online supplemental figures 3–5, online supplemental tables 4.1 and.2).

## Discussion

Reducing salt intake in China has the potential to substantially reduce CVD incidence and mortality in men and women of all ages across the country. As demonstrated in various parts of China, a reduction by 1 g/day in a year would be easily achievable.^5 21 22^ Should this very modest reduction be sustained, some 9M CVD events could be prevented by 2030; and if we account for the more prolonged effects of salt on blood pressure, this figure might increase to 10M. Achieving WHO's interim target of 30% reduction by 2025 and the Chinese government’s recommendation of bringing salt intake down to ≤5 g/day by 2030, representing average salt reductions of 3.2 g/day and 6 g/day, respectively, could prevent approximately 1.5 and 2 times more CVD events and deaths.

Our results are concordant with those of a previous simulation estimating the health impact of a 15% salt reduction in 23 low-income and middle-income countries (including China).^24^ Furthermore, when we accounted for the more prolonged effect of salt reduction on blood pressure, we obtained estimates of reductions in blood pressure and relative risk of CVD that correspond to what has been observed in countries with successful salt reduction programmes and 24-hour urinary sodium data, namely the UK and Finland.^19 25^

However, our estimates of CVD events and deaths prevented are greater than those of a previous projection by Wang *et al*.^26^ This discrepancy could be explained by Wang *et al*’s use of different salt reduction scenarios, notably affecting individuals with a high salt intake only (as opposed to the entire population); salt intake estimates derived from dietary assessment methods (which are notoriously unreliable in settings such as China, where most of the salt consumed comes from the discretionary salt^27^); smaller CVD risk reductions following SBP change based on a recalibration of the US-based Framingham Heart Study risk function^28^ (while we derived ours from a pooled analysis of 1.38 million participants in North America, Western Europe and Asia Pacific^20^); accounting for lag time (while we did not, due to the uncertainty around the time it takes for sustained reductions in SBP to reduce CVD risk) and obsolete CVD incidence rates (dating back to 1991–2009).^29^ A more in-depth discussion around CVD relative risks and incidence can be found in the online supplemental materials.

Our study has multiple strengths. First, we made use of the most up-to-date and robust data available. Baseline salt intake and the effect of salt reduction on blood pressure were derived from 24-hour urinary sodium excretions, that is, the most accurate method to assess salt intake.^6 13 19 27^ Salt intake and blood pressure data were extracted from the published baseline data of three large-scale cluster randomised controlled trials of over 5000 adult participants from six different provinces throughout China (Qinghai, Hebei, Heilongjiang, Sichuan, Jiangxi and Hunan), where stringent protocols were followed to ensure data collection quality.^6^ The parameter estimate of the relationship between salt reduction and SBP was drawn from a meta-regression of modest salt reduction trials lasting at least 4 weeks,^13^ so as to exclude trials bearing no relevance to public health (ie, trials of very short duration that consist of acute salt loading followed by severe salt restrictions, for example, from 20 g/day to less than 1 g/day of salt for only a few days). Nevertheless, it is still unlikely that salt reduction has exerted its maximal effect within 4–5 weeks (the average duration of the randomised trials included in the meta-regression)^30 31^ and we, therefore, for the first time to our knowledge, conducted additional analyses using parameter estimates from a population study to approximate the more prolonged effect of salt reduction on blood pressure over several years. Second, we modelled salt reductions that are highly relevant to policy-making, as they align with key national and international salt reduction targets, both in the extent of salt reduction and in time frame. Moreover, we modelled them as gradual percentage reductions, which is more likely to reflect the reality of salt reduction than a linear or an ‘overnight’ reduction. As existing models did not provide enough flexibility to suit the structure and parameterisation needed to meet our modelling objectives,^9–12^ a de novo model was built. Though not formally piloted, calibrated and validated, the model’s results were concordant with those of other major modelling studies^24^ as well as with empirical data from countries with successful salt reduction programmes.^19 25 32^

Due to scarce data, we were unable to model all health gains that would be expected from salt reduction. First, we did not account for the reduction in the risk for recurrent CVD events (ie, secondary prevention) and for diseases other than CVD, such as chronic kidney disease and gastric cancer, the rates of which are either increasing or already very high in China.^33^ Second, it has been suggested that higher salt intake levels were associated with a greater increase in blood pressure as one gets older.^34^ This means that in addition to lowering blood pressure immediately, salt reduction could also attenuate the rise in blood pressure associated with ageing. Taking this into account would have captured the full effect of salt reduction on blood pressure; however, there was insufficient data to quantify the association between salt intake and the rise in blood pressure with age. Third, although it has been shown that salt reduction in childhood leads to falls in blood pressure that could prevent hypertension and CVD in later life,^35^ we did not have sufficient data to include children in our model. Fourth, the proportion of haemorrhagic strokes is significantly higher in countries like China, and raised blood pressure is a stronger predictor of haemorrhagic than ischaemic strokes.^36 37^ The inclusion of Prospective Studies Collaboration participants (90% of whom from Europe, North America or Australia) in the pooling of relative risks of SBP change on CVD risk may have led to an underestimation of the potential impact of salt and SBP reduction on stroke incidence in China. It is, therefore, probable that we have underestimated the full potential of reducing salt intake in China.

The Chinese government’s action plan ‘Healthy China 2030’ includes nutritional recommendations to reduce the intake of salt, sugar and oil.^23^ This modelling study shows that salt reduction alone could bring enormous health benefits to the entire population of China. It is important to note that our estimates rely on salt reductions to not only be achieved, but also sustained over time, which may be a great challenge given the fast-changing dietary patterns seen in China given its rapid urbanisation. Most notably, the consumption of processed and out-of-home foods has increased in recent years and this trend is expected to continue.^8^ Processed foods in China have also been found to be saltier than in other countries.^38^ To anticipate this, it would be necessary to implement a strategy based on setting incremental salt targets for all manufactured foods in order to decrease salt content over the whole range of products from the food industry, as pioneered in the UK and successfully adopted by many countries, for example, Australia and South Africa.^39^ Nevertheless, most (70%–75%) of the salt consumed in China still comes from the salt added by the consumer during cooking.^8 40^ Health education can effectively lead to behaviour change, as shown in the trial in Northern China of a school-based programme, which was successful in reducing salt intake in both schoolchildren and their families.^21^ A scale-up study of this school-based programme is currently ongoing in other parts of China, with the aim of nationwide implementation if proved to be effective. Other trials, on low-sodium high-potassium salt substitutes, health education to home cooks and restaurant interventions are ongoing or have recently been completed, some of which have already shown promising results.^41^

The evidence for the substantial benefits of salt reduction in China is consistent and compelling. Achieving and sustaining population salt reduction in China could prevent millions of unnecessary cardiovascular events and deaths. Given the sheer size of the Chinese population, this would also bring major benefits to global health.

## Data Availability

Data are available on reasonable request. Requests for access to data and statistical code should be addressed to MT.
