# Supplementary material for: Reducing daily salt intake in China by 1 g could prevent almost 9 million cardiovascular events by 2030: a modelling study
Source: BMJ Nutr Prev Health. 2022 Aug 16;5(2):164–70. doi: 10.1136/bmjnph-2021-000408 (PMC9813635; doi:10.1136/bmjnph-2021-000408)
Supplement: Supplementary data [file bmjnph-2021-000408supp001.pdf]

SUPPLEMENTARY MATERIALS

Table of Contents

Baseline values 2
Population size 2
Generating values for levels of salt intake and systolic blood pressure 2
Baseline disease rates 3
Modelling salt reductions 4
Modelling health outcomes 5
Step 1: Modelling systolic blood pressure reduction 5
Step 2: Modelling cardiovascular disease outcomes 7
Uncertainty analysis 9
References for supplementary materials 29

List of tables

- Supplementary Table 1. Data sources
- Supplementary Table 2. Statistical distributions and parameters used in the uncertainty analysis
- Supplementary Table 3. Baseline disease rates used in the model
- Supplementary Table 4.1. Reduction in salt intake, systolic blood pressure, and CVD risk, events, deaths, and premature deaths, with effect size of salt reduction based on randomised trials
- Supplementary Table 4.2. Reduction in salt intake, systolic blood pressure, and CVD risk, events, deaths, and premature deaths, with effect size of salt reduction based on a population study (more prolonged effect)

Tan M, *et al. bmjnph* 2022; 5:e000408. doi: 10.1136/bmjnph-2021-000408

Baseline values

Population size

Population size was extracted from the latest governmental censuses.[1 2] Population subgroups were defined by 5-year age groups, sex, and geographical regions. Regions were defined as northern and southern China (Supplementary Figure 1), as distinct patterns in salt intake over time were found between those two regions.[3] The North-South demarcation was determined using a spatial analysis using geographic information system, following a model of climate-, geography-, and human-related indicators.[4]

Supplementary Figure 1. Definition of regions

White crosses indicate the provinces where the *"Action on Salt China"* study sites were located.

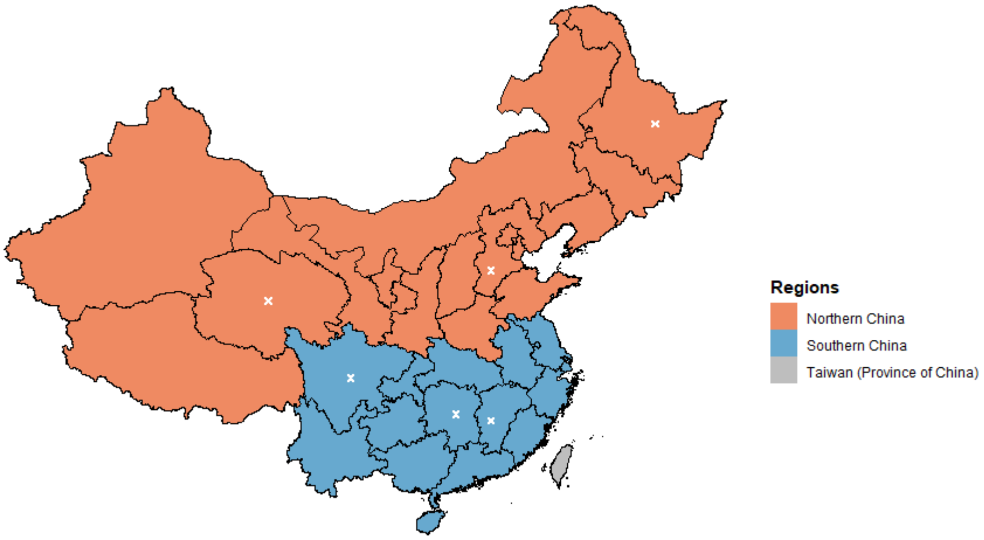

Generating values for levels of salt intake and systolic blood pressure

Each individual in our model was assigned a salt intake and a systolic blood pressure (SBP). This was done by generating values based on the means and covariance of salt intake and SBP (specific to each region-, age-, and sex-subgroup) from the published baseline results of "*Action on Salt China*", a programme of salt reduction in China consisting of three large-scale cluster randomised controlled trials (RCTs). Together, they involved 5353 adult participants from six different provinces throughout China (Qinghai, Hebei, Heilongjiang, Sichuan, Jiangxi, and Hunan; indicated with white crosses in Supplementary Figure 1), so as to represent the broad range of dietary habits and economic levels in the country. Participants were randomly selected from the general population using a two-stage sampling procedure.[5 6]

In the three *"Action on Salt China"* RCTs, salt intake was measured by 24h urinary sodium excretion, i.e. the most accurate method to assess salt intake.[7] The combined baseline data of the three RCTs, collected in September-November 2018, represent the largest collection of

Tan M, *et al. bmjnph* 2022; 5:e000408. doi: 10.1136/bmjnph-2021-000408

24h urinary sodium excretion ever conducted in China; and this collection followed a stringent protocol to ensure data quality.[6 8] All participants had one to two consecutive 24h urine sample(s) collected, and the completeness of the urine samples was determined using well-established criteria. Salt intake (g/d) was derived from the 24h urinary sodium excretion using standard conversion values (1 mmol sodium = 23 mg sodium, 1 g sodium = 2.5 g salt).[6 8]

As to SBP, the participants' blood pressure was measured at the baseline of the "*Action on Salt China"* RCTs by averaging the last two of three readings. Measurements were taken with validated automated electronic sphygmomanometers on the participants' right arm at 1-minute intervals, after they had rested in sitting position for about 10 minutes in a quiet room.[6 8]

Baseline disease rates

The use of disease rate estimates that were as recent as possible was crucial, as the burden of cardiovascular disease (CVD) in China has rapidly increased since 1990, and is now the leading cause of both death and premature death.[9] For example, the mortality rate of ischaemic heart disease (IHD) per 100,000 population is estimated to have increased significantly over the past three decades, from 52.2 (95% uncertainty interval [UI]: 49.2–55.6) in 1990 to 105.6 (95%UI: 101.0–110.8) in 2015.[10] Over that same time period, the incidence rates of both IHD and stroke have also risen steadily.[9]

In order to obtain incidence and mortality rates for IHD and stroke that were (i) recent and (ii) disaggregated by region-, age-, and sex- subgroups, a combination of different data sources was necessary. Although empirical data were preferred, they were often obsolete or unavailable (Supplementary Table 1).

For stroke, we obtained region-specific incidence and mortality rates from a nationally representative door-to-door survey conducted in 2013 in 31 provinces (n=480,687).[11] From this survey, we extracted the province-level incidence and mortality rates for stroke. We then determined the relative difference between the rates in each region and the average rates at the national level. These regional differences to the national mean rates were then applied to the more recent (2017) estimates of stroke incidence and mortality rates, which were reported at the national level and disaggregated by age and sex. These 2017 estimates were modelled by the Global Burden of Disease (GBD) collaborators using original data from national health surveillance programmes, vital registrations, governmental censuses, and hospital data (more details in Supplementary Table 1).

Tan M, *et al. bmjnph* 2022; 5:e000408. doi: 10.1136/bmjnph-2021-000408

For IHD, the latest nationally representative and empirical data for incidence rate come from the 1999-2000 follow-up of a cohort that started in 1991 (the China Hypertension Epidemiology Follow-up Study).[12] As these estimates are unlikely to reflect current disease rates, we used modelled data from the GBD study to obtain more recent estimates. The original data used by the GBD collaborators to estimate IHD rates in China are similar to those used to estimate stroke rates, and are presented in Supplementary Table 1. From a 2015 GBD publication, we obtained province-specific (but neither age- nor sex-disaggregated) mortality rate estimates, from which we determined the relative difference in the rates of each region with the national average rates. We then applied those regional differences to the 2017 national-level GBD estimates for incidence and mortality rates, which were disaggregated by age and sex (Supplementary Table 1).

All disease rates thus obtained and used in our model are presented for each region-, age-, and sex- subgroup in Supplementary Table 3.

Modelling salt reductions

Starting at the same time and with the same baseline levels of salt intake, three different salt reductions were modelled:

- A 1 g/d reduction to be achieved in a year;
- A 30% reduction to be achieved by 2025, following the World Health Organization's interim global target (modelled as a gradual 7% reduction per year);
- A reduction to ≤5 g/d by 2030, following the *"Healthy China 2030"* target (modelled as a gradual ~8% reduction per year).

In all cases, once the salt reduction was achieved, the reduced salt intake levels were then maintained for the rest of the simulation. All salt reductions were compared to a reference case without salt reduction (Supplementary Figure 2).

The mean salt intake levels in our simulations ranged from a maximum of 10.8 g/d to a minimum of 4.6 g/d. Within this range, the association between salt intake and blood pressure has been demonstrated to be linear,[13 14] and that between salt intake and mortality as well.[15]

Tan M, *et al. bmjnph* 2022; 5:e000408. doi: 10.1136/bmjnph-2021-000408

Supplementary Figure 2. Salt intake
Lines depict mean population salt intake (± standard deviation).

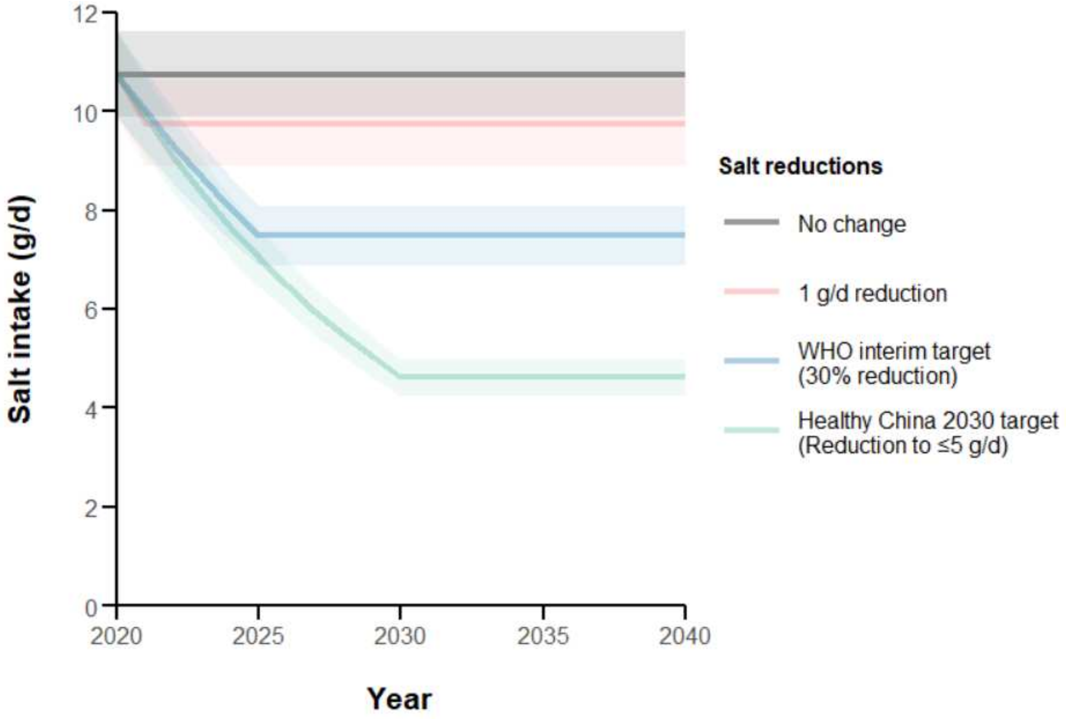

Modelling health outcomes

We estimated the impact of reducing salt intake on CVD incidence and mortality in China. The well-established causal relationships between salt intake, SBP, and CVD risk were reflected in our model by using a two-step process, whereby salt reduction led to SBP reduction, which in turn resulted in CVD risk reduction.[16] Each step is described below.

Step 1: Modelling systolic blood pressure reduction

Following common practice in salt reduction modelling,[17] we used SBP in our model, as SBP has been shown to be a better predictor of CVD risk than diastolic blood pressure.[18-22]

To describe the relationship between salt reduction and fall in SBP, we used the effect size estimate from He et al.'s systematic review and meta-analysis of randomised trials of salt reduction.[23] We used this source as it aimed to estimate the effect of a longer-term, modest salt reduction, and thus only included trials lasting at least 4 weeks. This therefore excluded very short (e.g. 3-5 days) trials of abrupt and severe salt restriction following acute salt loading, which are of no relevance to the public health recommendation for a modest salt reduction over a prolonged period of time. From He et al.'s systematic review and meta-analysis, we extracted the effect size of salt reduction on SBP from their meta-regression, as this analysis was adjusted for age and ethnic group and was reported for normotensive and hypertensive individuals separately.

Tan M, *et al. bmjnph* 2022; 5:e000408. doi: 10.1136/bmjnph-2021-000408

Similar effect sizes were found by Huang et al. in their systematic review and meta-analysis of randomised trials of salt reduction, published in 2020.[24] Although more recent than He et al.'s review, Huang et al.'s review included trials of very short duration, with more than half of their data points (77 out of 136) reporting interventions that lasted less than two weeks. A key finding of Huang et al.'s review is that such short-term studies underestimate the effect of salt reduction on blood pressure. In view of this, we derived our parameter estimate for the effect of salt reduction on SBP from He et al.'s review instead.

Nevertheless, the median duration of the trials included in He et al.'s meta-regression was of 4-5 weeks. As the full effect of salt reduction on blood pressure is unlikely to have been exerted over this time span,[25 26] we used a population study to estimate the more prolonged (over several years) effect of salt reduction on blood pressure in an additional analysis. The population study consisted of the analysis of nationally representative health and nutrition survey data as well as data from the Office for National Statistics for England and Wales.[27] Salt intake, as measured by the most accurate assessment method of 24h urinary sodium excretion, was shown to be reduced by 15% between 2003 and 2011 (from 9.5±0.2 to 8.1±0.2 g/d). Over those eight years, average SBP in individuals who were not on antihypertensive medication was found to have decreased by 2.7±0.34 mm Hg after adjusting for potential confounding factors (i.e. age, sex, ethnic group, household income, alcohol consumption, fruit and vegetable intake, and body mass index), suggesting the fall in SBP to be largely attributable to salt reduction. The effect size thus obtained was about double that from He et al.'s meta-regression of randomised trials: according to the effect size estimated from the population study, for each 1 g/d salt reduction, SBP would fall by 1.9 mm Hg (95% confidence interval [CI] 1.5 to 2.4); whereas the effect size from the randomised trials for the same amount of salt reduction was estimated at 1.0 mm Hg (95% CI 0.5 to 1.6).[23 27]

We used this population study in England because to the best of our knowledge, no such population study exists for any low- or middle-income country. Among high-income countries, the only other population study using nationally representative 24h urinary sodium data was conducted in Finland. This study showed that in Finland from 1972 to 2002, salt intake was reduced from about 14 g/d to about 9 g/d, and blood pressure fell by over 10 mm Hg.[28 29] Although salt reduction is likely to have played an important role in the fall in blood pressure (especially given the rising rates of obesity and alcohol consumption over that time period), the authors did not formally estimate the proportion of blood pressure and disease risk reduction attributable to salt reduction – thus precluding us from using deriving any effect size from their study.

In terms of ethnicity, a consistent finding is that salt reduction has a greater effect on the blood pressure of those belonging to non-Caucasian ethnic groups compared with those of Caucasian descent.[23 24] However, the majority of the non-Caucasian participants were of black ethnicity and there is scarce evidence on the effect size specific to individuals of Chinese descent. To date, only six trials of salt reduction were conducted with Chinese participants. Five of those trials consisted of non-controlled, non-randomised dietary feeding studies in which all participants were sequentially assigned to a low-salt diet, then to a high-salt diet. In those trials, the participants' salt intake was reduced by amounts ranging from -9.2 to -12.4 g/d, and this salt restriction was maintained for a week only. Such large and sudden salt reductions that bear no relevance to public health. The sixth trial was conducted on hypertensive patients with chronic kidney disease, and thus its findings cannot be used for

Tan M, *et al. bmjnph* 2022; 5:e000408. doi: 10.1136/bmjnph-2021-000408

the general population.[30] Given the lack of randomised trials of modest, longer-term salt reduction in Chinese participants, we made the conservative assumption that the effect of salt reduction on blood pressure in Chinese individuals would correspond to that found in studies with predominantly Caucasian participants.

Step 2: Modelling cardiovascular disease outcomes

Numerous randomised trials have shown the benefits of lowering blood pressure on CVD risk and thus demonstrate the causal relationship between SBP and CVD risk.[31] We used estimates from observational studies instead to extract the relative risks for IHD and stroke, as cohort studies are based on the participants' usual risk factor levels, as opposed to the effect of pharmacological interventions that could act through more pathways than risk factor reduction alone. For example, in addition to their beneficial effect via blood pressure reduction, calcium channel blockers confer greater protection for stroke, and beta-blockers have a special protective effect in preventing IHD events in individuals a few years after an acute myocardial infarction.[32] Nevertheless, both meta-analyses of randomised trials of blood pressure-lowering drugs and meta-analyses of cohort studies have found similar magnitudes of reductions in disease events for the same reduction in blood pressure.[32]

The relative risks for IHD and stroke were thus extracted from a pooled analysis of the Prospective Studies Collaboration (PSC)[18] and the Asia-Pacific Cohort Studies Collaboration (APCSC),[33] together representing a total of 99 cohorts with 1.38 million participants in North America, Western Europe, and the Asia-Pacific, totalling 65,000 CVD events and over 15 million person-years of follow-up. For the pooling of the PSC and the APCSC, the relative risks were adjusted for regression bias with age-specific correction factors and by accounting for the time between baseline SBP measurement and CVD event occurrence. Moreover, the use of individual data allowed for a consistent adjustment for confounders.[34] For our present study, the relative risks reported for ischaemic stroke and haemorrhagic stroke were pooled using random-effects meta-analysis.

The pooling of the PSC and APCSC showed that the proportional effect of SBP change on the relative risk for CVD declined with age, while being generally similar between men and women and between Western and Asian populations.[34]

The relative risks we extracted and used in our model are presented in the main manuscript, Table 1.

Tan M, *et al. bmjnph* 2022; 5:e000408. doi: 10.1136/bmjnph-2021-000408

Supplementary Table 1. Data sources

Model parameters		Data sources
Baseline values	Population size	Latest population census from the National Bureau of Statistics of China[1] and population estimates for the corresponding year from the Census and Statistics Department of the Government of the Hong Kong Special Administrative Region[2]
	Baseline salt intake and systolic blood pressure levels	Values generated using subgroup-specific means and covariance of salt intake and systolic blood pressure from the published baseline data of three cluster randomised controlled trials in six provinces throughout China (n=5353), as part of the "*Action on Salt China*" programme[5]
	Baseline disease incidence and mortality rates	A combination of different sources was necessary to obtain age-, sex-, and region-specific estimates.<br>- Stroke: Nationally representative door-to-door survey across 31 provinces of the prevalence, incidence, and mortality of stroke in China (n=480,687)[11]<br>- Ischaemic heart disease and stroke: Original data from sources including national health surveillance programmes (China Disease Surveillance Points, China Maternal and Child Health Surveillance System), vital registrations (Chinese Center for Disease Control and Prevention Cause of Death Reporting System, Hong Kong Vital Registration Death Data), governmental censuses (China Statistical Yearbook), and hospital data (China Hospital Inpatient Data) adapted by the Global Burden of Disease collaborators[10 35]
Effect estimates	Effect of salt reduction on systolic blood pressure	- Meta-regression of randomised trials of salt reduction, adjusted for age and ethnic group, and reported by blood pressure status (median trial duration: 4-5 weeks).[23]<br>- Population study of salt reduction (timespan observed: 8 years).[27]
	Effect of systolic blood pressure change on cardiovascular disease risk (relative risks)	Pooling of meta-analysis of epidemiological studies (Asia Pacific Cohort Studies Collaboration – APCSC; and the Prospective Studies Collaboration – PSC), representing a total of 1.38M participants with 65,000 cardiovascular disease events from 99 cohorts.[34]

There is considerable uncertainty around the time taken for sustained reduction in systolic blood pressure to reduce CVD risk. While some authors included various time lags in CVD risk reduction or reversal*,[36-38] others did not.[39-43] Notably, in their proportional multistate life-table model of the effect of salt reduction interventions on CVD in the Australian population, Cobiac et al. found similar outcomes with and without incorporating a 3-year-time lag for full reversal of stroke risk and a 3-year-time lag for a two-thirds reversal of heart risk, with the remaining heart disease risk reversed over 7 subsequent years.[44] In order to avoid introducing another source of uncertainty and undue complexity in this present study, we assumed that the effect of blood pressure reduction on CVD risk is fully realised within one year. This is in line with the findings of a review of randomised trials evaluating the effects of blood-pressure-lowering drugs on heart disease and stroke events.[32]

Uncertainty analysis

We conducted a Monte Carlo analysis with 5000 iterations to quantify the uncertainty in the modelled results due to uncertainty around the model parameters. Uncertainty around the following parameters were included in the analysis: baseline salt intake and systolic blood pressure estimated from the *"Action on Salt China"* trials, effect of salt reduction on SBP estimated by the meta-regression and the population-based study, relative risks of IHD and stroke following blood pressure reduction estimated by pooling of cohort studies. The prior distributions were estimated using data from the source material (Supplementary Table 2).

At each iteration, the model parameters were drawn randomly from the specified distributions. We assumed Poisson distributions for disease rates, log-normal distributions for relative risks, and normal distributions for all other parameters. The salt reductions were then modelled using this random set of model parameters. Results for the iterations were saved, and uncertainty intervals for the results were based on the 2.5th and 97.5th percentile of the results across the 5000 iterations.

* In this context, risk reversal is defined as the diminution of excess CVD risk and raised blood pressure, down to the CVD risk levels of individuals who never had raised blood pressure.

Tan M, *et al. bmjnph* 2022; 5:e000408. doi: 10.1136/bmjnph-2021-000408

Supplementary Table 2. Statistical distributions and parameters used in the uncertainty analysis

Inputs	Distributions	Parameters	Source
Baseline salt intake	Normal	Mean and SD of 24h urinary sodium excretion	Baseline results of the *"Action on Salt China"* trials[5]
Baseline systolic blood pressure levels	Normal	Mean and SD of systolic blood pressure	Baseline results of the *"Action on Salt China"* trials[5]
Baseline disease incidence and mortality rates	Poisson	Lambda = disease rate per 100,000 person-years	Nationally representative door-to-door survey[11] and Global Burden of Disease estimates[10 35]
Effect of salt reduction on systolic blood pressure	Normal	Mean = regression coefficient<br><br>SD = SE of the coefficient	Meta-regression of randomised trials of salt reduction[23] and population study[27]
Effect of systolic blood pressure change on cardiovascular disease risk (relative risks)	Lognormal	Mean on the log scale = natural logarithm of the relative risk<br><br>SD on the log scale = natural logarithm of the SE (estimated from 95% confidence interval)	Pooled cohort studies[34]

SD = standard deviation; SE = standard error.

Tan M, *et al. bmjnph* 2022; 5:e000408. doi: 10.1136/bmjnph-2021-000408

Supplementary Table 3. Baseline disease rates used in the model

Abbreviations: IHD = ischaemic heart disease, SD = standard deviation, UI = uncertainty interval.

Region	Age groups (years)	Sex	IHD incidence rate per 100,000 person-years Median (95% UI)	IHD mortality rate per 100,000 person-years Median (95% UI)	Stroke incidence rate per 100,000 person-years Median (95% UI)	Stroke mortality rate per 100,000 person-years Median (95% UI)
**Northern**	35–39	Women	19.5 (8.5–38.2)	5.4 (4.3–6.7)	70.1 (33.4–114.8)	22.7 (4.7–48)
**Northern**	40–44	Women	35.7 (21.7–55.7)	9.8 (8–12.1)	404 (270.2–544.2)	117.1 (56.9–185.8)
**Northern**	45–49	Women	59.7 (33.7–95.8)	22.3 (18.2–27.2)	404 (270.2–544.2)	117.1 (56.9–185.8)
**Northern**	50–54	Women	93.9 (59.5–138.6)	38.6 (31.6–47.1)	1984.5 (1439.5–2505.1)	429.3 (239.3–624.4)
**Northern**	55–59	Women	138.1 (75.9–217.7)	53 (43.5–64.5)	1984.5 (1439.5–2505.1)	429.3 (239.3–624.4)
**Northern**	60–64	Women	233.4 (156.6–333.6)	139.3 (114.9–167.8)	4678.7 (3466.7–5796.5)	932.1 (543–1312.6)
**Northern**	65–69	Women	378.6 (241.5–577.6)	291.4 (239.2–351.8)	4678.7 (3466.7–5796.5)	932.1 (543–1312.6)
**Northern**	70–79	Women	591 (419.5–839.4)	494.3 (405.2–596.3)	7179 (5289.9–8938.1)	1540.4 (890.7–2182)
**Northern**	35–39	Men	25.3 (11.9–46.6)	18.9 (15.4–23.1)	96.2 (50.5–150.5)	33.3 (9.7–64.9)
**Northern**	40–44	Men	50.4 (31.4–76.4)	34.7 (28.4–42.2)	549.4 (377–725.7)	175.4 (91.3–267.3)
**Northern**	45–49	Men	88.9 (52.7–137.5)	66.3 (54.2–80.3)	549.4 (377–725.7)	175.4 (91.3–267.3)
**Northern**	50–54	Men	149.3 (97.9–214)	106.9 (87.6–129.3)	2552.5 (1868.4–3196.7)	666.6 (385.2–943.9)
**Northern**	55–59	Men	231.8 (138.2–353.5)	134.4 (111–161.5)	2552.5 (1868.4–3196.7)	666.6 (385.2–943.9)
**Northern**	60–64	Men	362.2 (247.3–508.2)	271.7 (224.3–326)	5762.5 (4288.8–7110.7)	1146 (674.3–1600.2)
**Northern**	65–69	Men	538.2 (349.8–810.8)	478.7 (395.5–573.5)	5762.5 (4288.8–7110.7)	1146 (674.3–1600.2)
**Northern**	70–79	Men	777.9 (552.2–1098.9)	698.2 (577.5–835.4)	9180.1 (6807.2–11365.6)	1875.3 (1094.6–2635.3)
**Southern**	35–39	Women	13.3 (5.8–25.9)	3.7 (3–4.5)	63.2 (33.4–97.3)	19 (5.1–34.8)
**Southern**	40–44	Women	24.3 (14.9–37.8)	6.7 (5.5–8.2)	364.1 (270.3–461.3)	98 (62.2–134.6)
**Southern**	45–49	Women	40.6 (23.1–65)	15.1 (12.5–18.4)	364.1 (270.3–461.3)	98 (62.2–134.6)
**Southern**	50–54	Women	63.8 (40.8–94)	26.2 (21.6–31.9)	1787.2 (1439.1–2122.2)	359.2 (261.2–452.3)
**Southern**	55–59	Women	93.7 (51.9–147.3)	36 (29.8–43.7)	1787.2 (1439.1–2122.2)	359.2 (261.2–452.3)
**Southern**	60–64	Women	158.6 (107.3–226.1)	94.6 (78.7–113.7)	4214.2 (3466.3–4911.3)	780 (592.7–950.8)
**Southern**	65–69	Women	257.1 (165.5–391.3)	197.9 (163.9–238.3)	4214.2 (3466.3–4911.3)	780 (592.7–950.8)
**Southern**	70–79	Women	401.4 (287.4–568.8)	335.7 (277.6–404)	6465.4 (5288.6–7572.1)	1289 (972.1–1580.4)
**Southern**	35–39	Men	17.2 (8.1–31.6)	12.9 (10.6–15.6)	86.6 (50.4–127.4)	27.9 (10.6–47.1)
**Southern**	40–44	Men	34.3 (21.5–51.8)	23.6 (19.5–28.6)	494.3 (376.6–614.2)	147 (100.1–193.8)
**Southern**	45–49	Men	60.5 (36.1–93.3)	45.1 (37.2–54.5)	494.3 (376.6–614.2)	147 (100.1–193.8)
**Southern**	50–54	Men	101.5 (67.1–145.1)	72.7 (60.1–87.7)	2294 (1864.3–2702.2)	558.2 (421.5–683.7)
**Southern**	55–59	Men	157.3 (94.6–239.3)	91.2 (76–109.3)	2294 (1864.3–2702.2)	558.2 (421.5–683.7)

BMJ Publishing Group Limited (BMJ) disclaims all liability and responsibility arising from any reliance placed on this supplemental material which has been supplied by the author(s)

**Southern**	60–64	Men	246 (169.5–344.4)	184.5 (153.7–220.9)	5177.9 (4278.6–6009.6)	959.4 (737.8–1159)
**Southern**	65–69	Men	365.5 (239.7–549.4)	325.1 (271–388.6)	5177.9 (4278.6–6009.6)	959.4 (737.8–1159)
**Southern**	70–79	Men	528.3 (378.3–744.6)	474.2 (395.7–566)	8247.4 (6789.9–9604)	1569.9 (1197.5–1908.4)

Supplementary Figure 3. Number of CVD events and deaths prevented with different salt reductions, by sex, estimating the effect of salt reduction on blood pressure from (A) randomised trials and (B) a UK-based population study

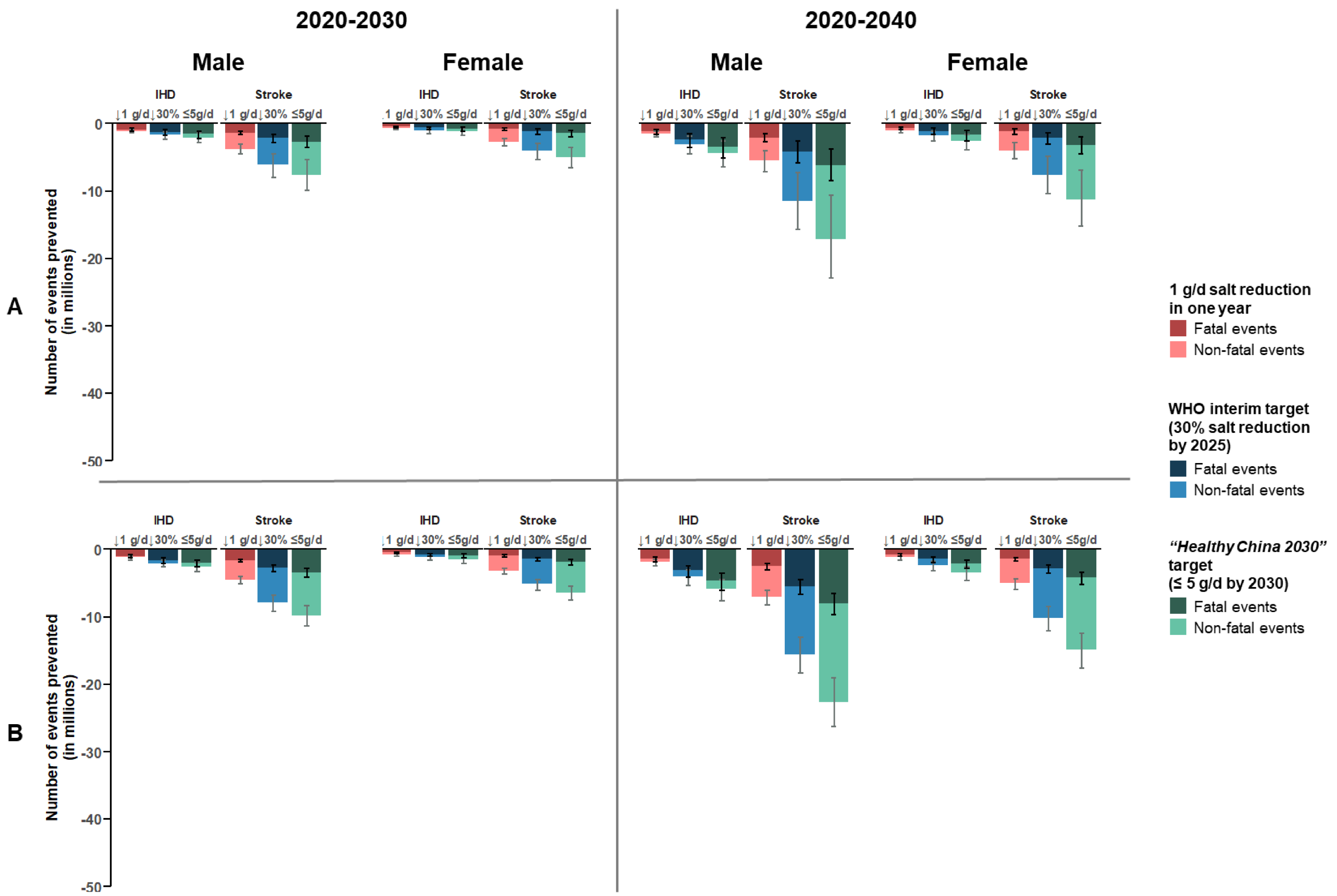

Supplementary Figure 4. Number of CVD events and deaths prevented with different salt reductions, by region, estimating the effect of salt reduction on blood pressure from (A) randomised trials and (B) a UK-based population study

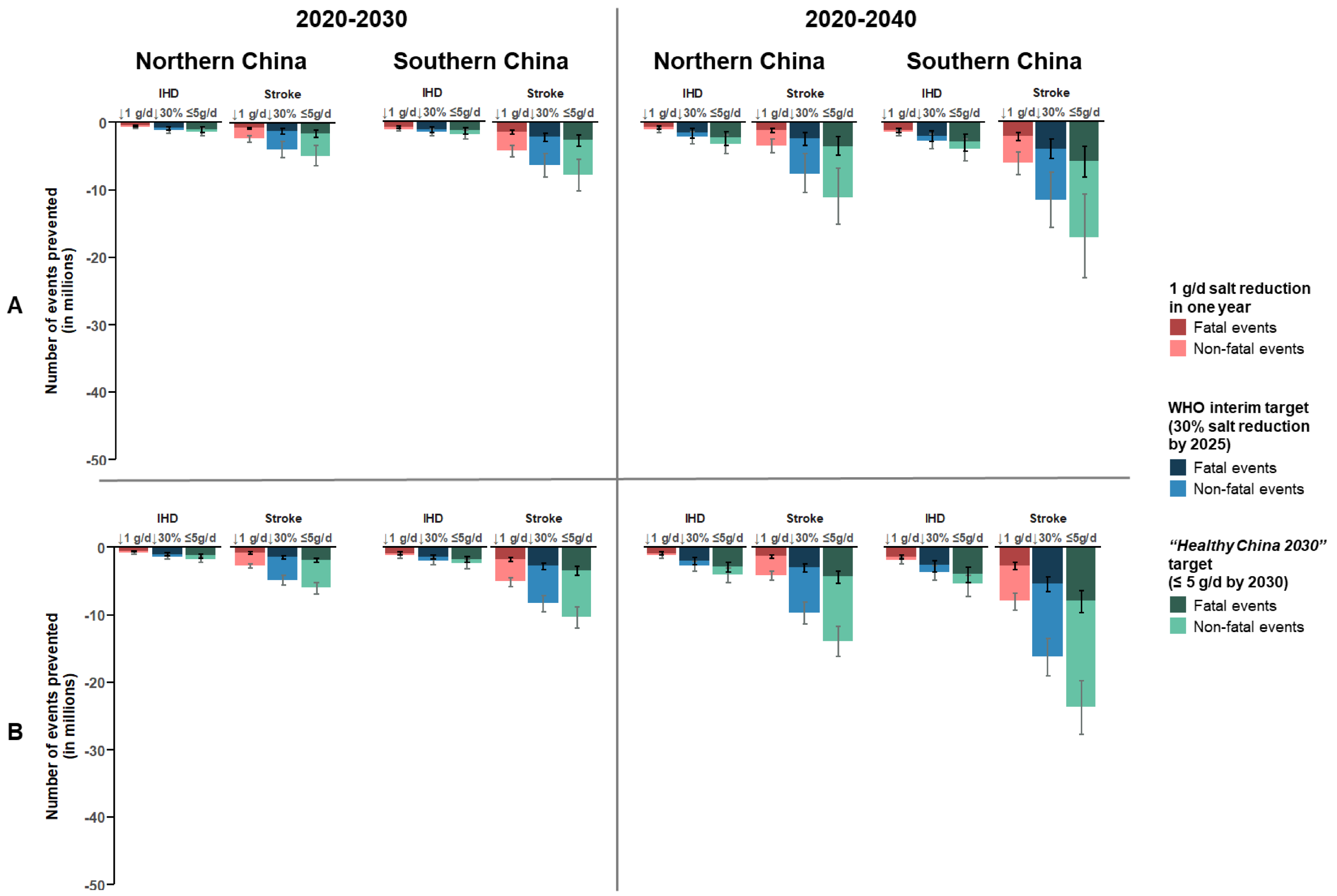

Supplementary Figure 5. Number of CVD events and deaths prevented with different salt reductions, by age group, estimating the effect of salt reduction on blood pressure from (A) randomised trials and (B) a UK-based population study

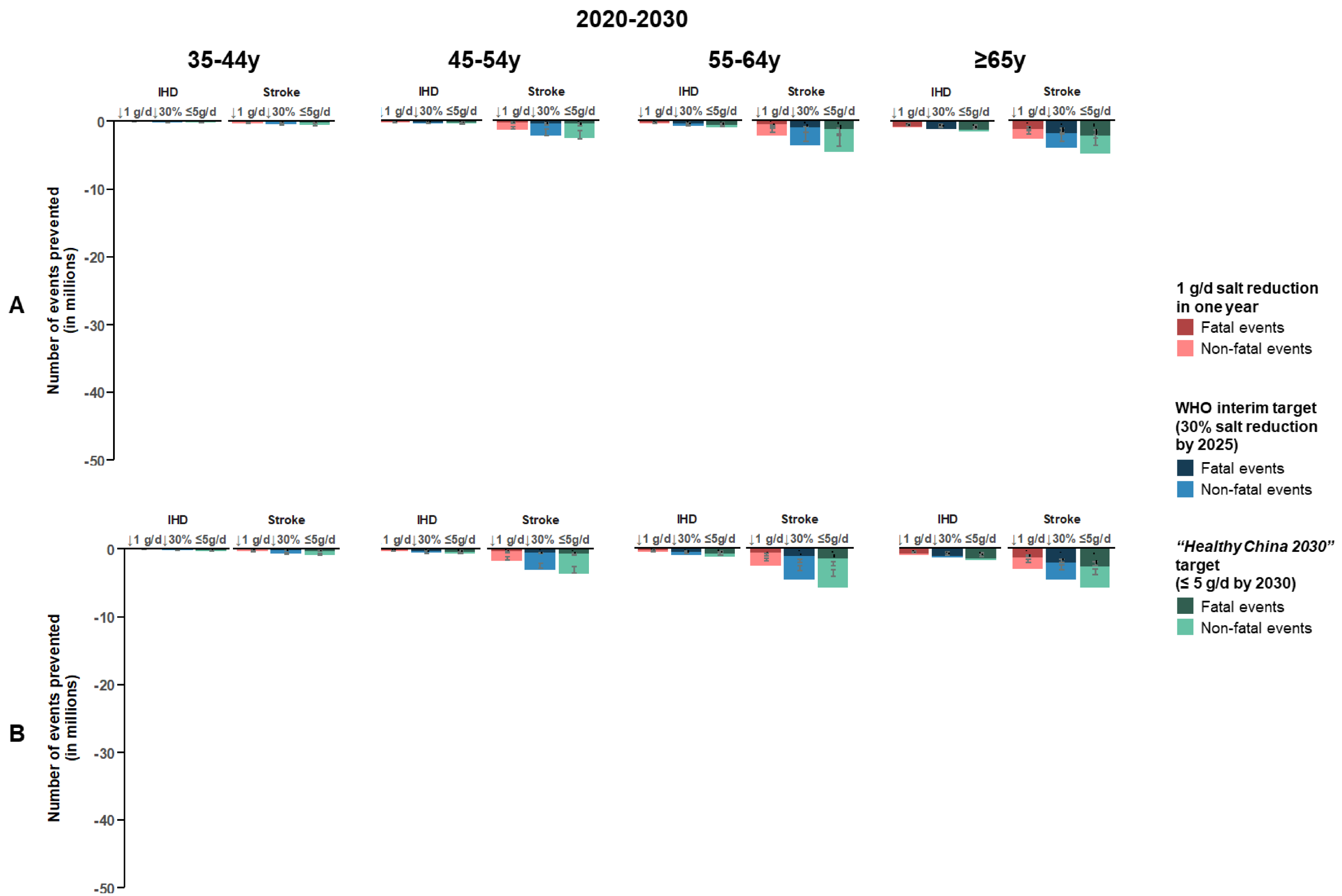

Supplementary Figure 5. *(continued)*

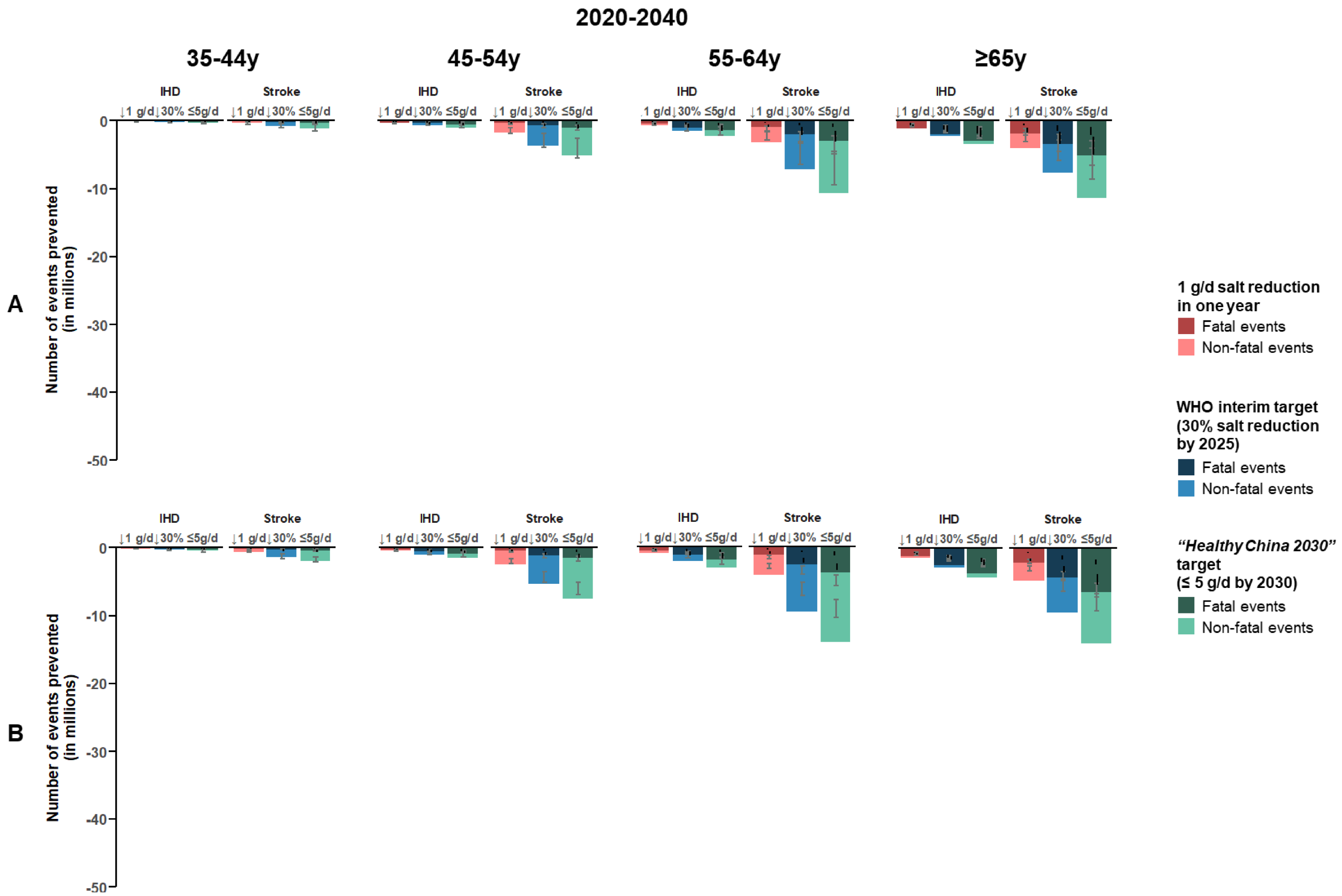

Tan M, *et al. bmjnph* 2022; 5:e000408. doi: 10.1136/bmjnph-2021-000408

Supplementary Table 4.1. Reduction in salt intake, systolic blood pressure, and CVD risk, events, deaths, and premature deaths, with effect size of salt reduction based on randomised trials

All results are reported in median (95% uncertainty interval). The changes reported for salt intake and SBP are absolute reductions; those reported for disease risk are relative reductions. Abbreviations: IHD = ischaemic heart disease, SBP = systolic blood pressure.

Region	Age groups (years)	Sex	Salt reductions	↓ salt intake (g/d)	↓ SBP (mm Hg)	↓ IHD risk (%)	↓ stroke risk (%)	↓ IHD events by 2030 (n)	↓ stroke events by 2030 (n)	↓ IHD deaths by 2030 (n)	↓ stroke deaths by 2030 (n)	↓ IHD events by 2040 (n)	↓ stroke events by 2040 (n)	↓ IHD deaths by 2040 (n)	↓ stroke deaths by 2040 (n)
**Northern**	35–39	Women	1 g/d in a year	1 (1–1)	0.8 (0.3–1.3)	3.8 (7.5–1.2)	5.5 (9.2–1.9)	4507 (7842–2507)	6057 (8725–3511)	1252 (2521–452)	712 (1663–0)	5504 (10531–3160)	7595 (11921–4534)	1569 (3253–518)	910 (2141–0)
**Northern**	35–39	Men	1 g/d in a year	1 (1–1)	0.9 (0.4–1.3)	4.2 (7.9–1.7)	6 (9.4–2.4)	6493 (10075–3663)	9696 (13845–6131)	4510 (7547–2415)	2132 (3966–903)	8200 (12935–4524)	12629 (19078–7187)	5625 (9807–2753)	2794 (5481–1210)
**Northern**	40–44	Women	1 g/d in a year	1 (1–1)	0.8 (0.3–1.3)	3.9 (7.7–1.2)	5.6 (8.9–2.1)	9068 (13365–5574)	33060 (43622–24614)	2435 (4479–1115)	7981 (12438–5420)	11414 (18763–6537)	45414 (65345–29750)	3219 (6010–1410)	10802 (18105–6592)
**Northern**	40–44	Men	1 g/d in a year	1 (1–1)	0.9 (0.4–1.3)	4.1 (7.8–1.5)	5.8 (9.1–2.4)	13362 (18082–9940)	51552 (63930–39494)	8893 (11827–5802)	11978 (17429–8114)	17069 (26370–12059)	71419 (96419–49587)	11537 (17317–7266)	16754 (25785–10767)
**Northern**	45–49	Women	1 g/d in a year	1 (1–1)	1 (0.5–1.4)	3.8 (8.2–2.1)	5.8 (8.5–3.2)	17052 (24843–12726)	37889 (46335–30884)	6419 (9673–4047)	9537 (13068–6797)	21140 (33677–14733)	50526 (65540–38226)	8068 (12741–4870)	12626 (18217–7951)
**Northern**	45–49	Men	1 g/d in a year	1 (1–1)	1.1 (0.6–1.5)	4.4 (8.9–2.3)	6.4 (9.3–3.6)	27778 (35163–21828)	60272 (75637–48161)	20674 (26990–16127)	14576 (18555–10404)	35015 (48145–26962)	81606 (107692–61616)	26308 (36520–19189)	19571 (26668–13544)
**Northern**	50–54	Women	1 g/d in a year	1 (1–1)	1.1 (0.6–1.5)	4.8 (8–2.3)	6.5 (9–3.5)	27800 (39648–20875)	143084 (168712–115768)	11237 (16699–7421)	27093 (36289–20984)	34897 (54740–25675)	192023 (238281–142029)	14347 (22806–9283)	36596 (50391–26326)
**Northern**	50–54	Men	1 g/d in a year	1 (1–1)	1.3 (0.7–1.9)	5.3 (9.1–2.7)	7.3 (10.7–4)	46412 (61167–37698)	237402 (287474–190352)	33438 (44140–26996)	39789 (51004–31782)	60467 (86502–44914)	322240 (416943–236457)	43342 (62073–33209)	54828 (74161–40000)
**Northern**	55–59	Women	1 g/d in a year	1 (1–1)	1.4 (0.8–2.2)	4.9 (8.2–2.6)	6.7 (10–3.9)	29863 (41795–23056)	106966 (131887–81706)	11507 (16499–7987)	20952 (26808–15563)	41976 (63932–30170)	158849 (209929–111993)	16473 (25210–10187)	31234 (42386–21646)
**Northern**	55–59	Men	1 g/d in a year	1 (1–1)	1.4 (0.8–2.1)	4.8 (8–2.5)	6.6 (9.9–3.8)	50544 (69215–40154)	167985 (210159–133105)	29502 (39005–22599)	29153 (37843–21882)	71445 (107580–51326)	248483 (334467–180888)	41312 (59433–28885)	43576 (59863–30601)
**Northern**	60–64	Women	1 g/d in a year	1 (1–1)	1.5 (0.8–2.3)	5.4 (8.8–3)	7.1 (10.8–3.8)	50823 (64962–41137)	226787 (282579–182380)	30818 (39306–24645)	48358 (60701–36973)	75418 (105733–55596)	359042 (486869–253218)	46260 (63926–33306)	76819 (102399–51971)
**Northern**	60–64	Men	1 g/d in a year	1 (1–1)	1.5 (0.8–2.3)	5.3 (8.7–3)	7.1 (10.7–3.8)	80047 (102395–64919)	284870 (348836–220920)	60306 (75672–48438)	102185 (127656–81291)	118128 (170149–89134)	450413 (599700–307773)	89066 (121420–65223)	161150 (214285–111056)
**Northern**	65–69	Women	1 g/d in a year	1 (1–1)	1.7 (0.8–2.7)	4.6 (7.6–2.3)	6 (9.7–3.2)	61391 (77078–49323)	167066 (207911–134835)	47512 (61361–39497)	35974 (46709–27996)	93327 (131464–66418)	270058 (375056–191229)	72259 (104341–52187)	58897 (83425–40805)
**Northern**	65–69	Men	1 g/d in a year	1 (1–1)	1.6 (0.8–2.4)	4.4 (6.9–2.3)	5.6 (8.8–3.1)	87207 (105880–72321)	203029 (249753–162522)	77982 (95071–63465)	74220 (89312–61278)	131453 (172843–95494)	323480 (438051–228791)	116043 (154735–83251)	117090 (156301–85565)

**Northern**	70–79	Women	1 g/d in a year	1 (1–1)	1.6 (0.8–2.6)	4.6 (7–2.3)	5.7 (9.2–3)	67016 (83620–53904)	191604 (249200–145202)	55653 (71050–43108)	94404 (120645–69075)	105654 (144040–74494)	317300 (453700–209439)	88296 (121254–59183)	156213 (218921–100073)
**Northern**	70–79	Men	1 g/d in a year	1 (1–1)	1.5 (0.8–2.4)	4.3 (6.5–2.3)	5.4 (8.6–3.1)	86275 (107510–71767)	231874 (299391–185006)	76713 (93678–63965)	137279 (176483–105853)	134256 (181873–98100)	378856 (537251–267631)	119068 (157952–88667)	224846 (316623–155505)
**Southern**	35–39	Women	1 g/d in a year	1 (1–1)	0.8 (0.3–1.3)	3.9 (7.5–1.3)	5.6 (9.2–2)	6898 (12542–3810)	11165 (16790–4889)	2114 (4674–459)	1196 (3116–0)	8676 (16385–4581)	14734 (23204–6237)	2678 (5995–522)	1549 (4411–0)
**Southern**	35–39	Men	1 g/d in a year	1 (1–1)	0.8 (0.3–1.3)	4 (7.6–1.4)	5.6 (9.3–2.2)	9960 (15475–5430)	18038 (27085–10424)	7401 (13121–2957)	4110 (8913–1770)	12530 (20006–6236)	23403 (37006–13046)	9165 (17378–3576)	5382 (11834–2225)
**Southern**	40–44	Women	1 g/d in a year	1 (1–1)	0.8 (0.3–1.3)	3.9 (7.6–1.1)	5.5 (8.8–2)	12955 (19055–8208)	60298 (86953–43427)	3437 (6616–1119)	15855 (24907–9986)	16763 (26502–10474)	83436 (129700–50896)	4287 (9399–1444)	21689 (35784–12313)
**Southern**	40–44	Men	1 g/d in a year	1 (1–1)	0.9 (0.4–1.3)	4.1 (7.9–1.6)	5.9 (9.2–2.6)	20715 (30077–11575)	100096 (126997–71472)	14494 (21597–8495)	24669 (35770–16289)	26455 (42071–14078)	139328 (187457–87969)	19300 (30599–9770)	34333 (50918–20901)
**Southern**	45–49	Women	1 g/d in a year	1 (1–1)	0.9 (0.4–1.3)	3.5 (7.4–1.4)	5 (7.9–2.2)	26268 (37593–17761)	67230 (85039–49112)	9376 (14246–4644)	18955 (26622–13298)	32405 (49283–20427)	87778 (119192–57248)	11708 (18890–5712)	24892 (35666–16433)
**Southern**	45–49	Men	1 g/d in a year	1 (1–1)	1 (0.5–1.4)	3.7 (8–1.9)	5.6 (8.4–3)	41613 (54054–31945)	110707 (139671–91024)	31174 (48162–20756)	29558 (37696–21535)	51696 (73259–37599)	145994 (195700–108983)	39208 (67588–23526)	38904 (51453–26518)
**Southern**	50–54	Women	1 g/d in a year	1 (1–1)	1 (0.5–1.4)	4.4 (7.5–2)	5.9 (8.3–3)	40444 (54084–30038)	255313 (302620–207202)	17153 (24191–9827)	54508 (67865–40164)	50233 (73537–36043)	337313 (420686–251843)	21588 (31127–11826)	72917 (93287–48419)
**Southern**	50–54	Men	1 g/d in a year	1 (1–1)	1.1 (0.6–1.6)	5 (8.2–2.4)	6.7 (9.5–3.6)	72278 (90696–56083)	444214 (527256–343204)	51045 (66145–39766)	82415 (105899–62571)	92373 (126118–66281)	596011 (750138–429677)	66368 (90370–46468)	111481 (147808–78495)
**Southern**	55–59	Women	1 g/d in a year	1 (1–1)	1.2 (0.7–1.8)	4.2 (6.9–2.3)	5.9 (8.6–3.3)	40374 (57564–32062)	186026 (229625–154396)	15854 (23425–10948)	38977 (49865–29315)	56486 (85938–41688)	269831 (357002–207160)	22112 (33847–14460)	56239 (74994–39722)
**Southern**	55–59	Men	1 g/d in a year	1 (1–1)	1.2 (0.7–1.7)	4.1 (6.5–2.2)	5.6 (8–3)	74908 (94322–55854)	299176 (373146–243416)	41476 (57757–29757)	55367 (73753–40271)	102915 (135691–70277)	432588 (570140–317086)	57048 (83950–38226)	80219 (111210–54238)
**Southern**	60–64	Women	1 g/d in a year	1 (1–1)	1.3 (0.7–2)	4.7 (7.3–2.8)	6.2 (9.1–3.5)	72428 (93033–57684)	403134 (465689–321370)	44325 (56420–34894)	90407 (113316–73538)	104669 (143892–77420)	619179 (759191–443906)	63782 (86800–46792)	139615 (185054–102265)
**Southern**	60–64	Men	1 g/d in a year	1 (1–1)	1.3 (0.8–2)	4.8 (7.6–2.9)	6.4 (9.4–3.5)	121692 (153165–97991)	528429 (640069–426959)	89877 (113645–74355)	206174 (249398–170272)	175966 (240107–131622)	808942 (1062963–584043)	131477 (177830–100373)	315737 (418329–233110)
**Southern**	65–69	Women	1 g/d in a year	1 (1–1)	1.5 (0.8–2.2)	4 (6.3–2.2)	5.2 (8.2–3)	89140 (110056–75280)	290820 (365575–240156)	70174 (86920–54851)	67254 (86767–55677)	131624 (175765–100749)	457193 (629291–334554)	102067 (140174–72488)	104778 (148222–77347)
**Southern**	65–69	Men	1 g/d in a year	1 (1–1)	1.3 (0.7–1.9)	3.5 (5.4–2.1)	4.6 (7–2.8)	129486 (150478–107726)	368489 (446656–308088)	112852 (137962–100060)	143741 (170185–116700)	187014 (229455–141058)	563994 (744106–423805)	162383 (214570–134940)	219287 (284542–158789)
**Southern**	70–79	Women	1 g/d in a year	1 (1–1)	1.4 (0.8–2.2)	4 (6–2.3)	5 (8–3.1)	97235 (120627–80117)	340968 (429003–278235)	80384 (101463–65934)	178859 (229577–146753)	148259 (200053–109902)	550153 (757135–407040)	122879 (167098–91073)	288501 (409067–214950)
**Southern**	70–79	Men	1 g/d in a year	1 (1–1)	1.4 (0.8–2.2)	4 (6–2.4)	5.1 (8–3.1)	133578 (159196–110200)	437572 (545295–358581)	118950 (143540–97781)	277926 (340737–225652)	203410 (264351–151398)	708301 (963538–522746)	184296 (238795–135180)	447987 (605267–327253)

**Northern**	35–39	Women	30% by 2025	3.4 (3.4–3.4)	2.7 (0.9–4.3)	12.2 (22.9–4)	17.3 (27.6–6.3)	6603 (13065–3881)	9224 (14851–5228)	1865 (3982–570)	1117 (2670–0)	10154 (20837–5757)	14934 (24846–7596)	2827 (6214–795)	1774 (4441–0)
**Northern**	35–39	Men	30% by 2025	4.1 (4.1–4.1)	3.6 (1.6–5.4)	16.1 (28.6–6.7)	22.4 (33.3–9.7)	11047 (17995–5909)	17462 (26915–8984)	7507 (13708–3462)	3868 (7776–1685)	17747 (31039–8300)	28460 (45411–13112)	11594 (23361–5306)	6435 (13286–2523)
**Northern**	40–44	Women	30% by 2025	3.5 (3.5–3.5)	2.9 (1.1–4.6)	13.1 (24.6–4.3)	18.3 (28–7.3)	13071 (21987–7235)	52867 (76482–32865)	3716 (6997–1589)	12653 (21190–7332)	22394 (39862–10581)	91715 (144543–47426)	5829 (12765–2318)	22377 (39714–10802)
**Northern**	40–44	Men	30% by 2025	4 (4–4)	3.4 (1.5–5.3)	15.4 (28–6)	21.4 (31.9–9.5)	20976 (33421–13272)	89050 (122005–57842)	14032 (21884–8485)	20934 (32642–13453)	36180 (62812–19095)	162466 (235565–88552)	24252 (40340–12256)	38086 (62454–21324)
**Northern**	45–49	Women	30% by 2025	3.5 (3.5–3.5)	3.5 (1.9–4.8)	12.8 (25.7–7.1)	18.7 (26.8–10.8)	23865 (38753–16169)	58214 (75640–42948)	9052 (14586–5410)	14494 (21027–8726)	38178 (67250–23222)	99378 (137023–67743)	14588 (25261–7643)	24672 (37849–12980)
**Northern**	45–49	Men	30% by 2025	4.1 (4.1–4.1)	4.6 (2.5–6.3)	16.7 (31.7–8.9)	23.7 (32.8–13.9)	42816 (60418–31318)	101890 (135919–74624)	32024 (45956–22148)	24361 (33665–16709)	70640 (109364–47097)	180854 (252268–120278)	53497 (83571–33753)	43363 (62867–28590)
**Northern**	50–54	Women	30% by 2025	3.5 (3.5–3.5)	3.9 (2.1–5.3)	15.8 (25.2–7.7)	20.6 (27.9–11.5)	41854 (67235–29967)	233616 (292924–167093)	17451 (27941–10980)	44515 (61923–31211)	66562 (115415–44662)	390243 (508322–253630)	27502 (47267–16283)	73900 (106964–47481)
**Northern**	50–54	Men	30% by 2025	4.2 (4.2–4.2)	5.3 (3–7.9)	20.5 (33.1–11)	27.3 (37.8–15.9)	81963 (119995–56885)	444102 (578747–309582)	58443 (86160–43871)	75506 (102841–53660)	136861 (213082–85747)	768130 (1039636–492282)	98238 (151705–66804)	130490 (184954–86566)
**Northern**	55–59	Women	30% by 2025	3.3 (3.3–3.3)	4.6 (2.7–7.1)	15.3 (24.6–8.3)	20.5 (29.3–12.2)	50996 (77832–35393)	193342 (254012–134243)	19909 (30687–11894)	37999 (51379–25870)	90233 (144532–57197)	353895 (482833–229648)	35095 (56391–19629)	69333 (97302–44286)
**Northern**	55–59	Men	30% by 2025	3.8 (3.8–3.8)	5.3 (3–8.2)	17.2 (27.5–9.4)	22.9 (32.9–13.7)	95625 (144925–64409)	334461 (449329–236637)	54636 (80267–36698)	58767 (80516–39722)	170613 (276936–105116)	618041 (862960–410625)	97287 (151064–59720)	108969 (154131–67974)
**Northern**	60–64	Women	30% by 2025	3.5 (3.5–3.5)	5.2 (2.8–8)	17.3 (27.2–9.9)	22.6 (32.5–12.5)	82089 (114258–60059)	390011 (518806–274700)	50322 (69056–35930)	83395 (109126–56415)	160956 (241762–106662)	803697 (1135515–508466)	99163 (146023–63879)	172338 (238707–105329)
**Northern**	60–64	Men	30% by 2025	4 (4–4)	6 (3.3–9.2)	19.7 (30.7–11.4)	25.6 (36.5–14.4)	140154 (201016–104124)	535141 (700533–360538)	105835 (144240–75340)	191386 (252610–129545)	284881 (438933–193956)	1134075 (1561361–692263)	216256 (307672–136596)	406132 (554299–242765)
**Northern**	65–69	Women	30% by 2025	3 (3–3)	5.1 (2.4–7.9)	13.1 (20.8–6.8)	16.7 (26.1–9.1)	90719 (124945–65499)	260855 (349837–187759)	70199 (99245–51576)	56719 (78108–40060)	182493 (274793–115811)	545842 (798208–349751)	141923 (217651–88348)	119440 (177269–74633)
**Northern**	65–69	Men	30% by 2025	3.8 (3.8–3.8)	5.9 (3–9.2)	15.4 (23.5–8.3)	19.4 (29.3–11.1)	145978 (191524–104065)	359517 (479969–252149)	128730 (171663–90793)	130101 (171114–94298)	301672 (427970–188148)	777466 (1110862–487243)	266518 (381210–163587)	282517 (397286–182182)
**Northern**	70–79	Women	30% by 2025	2.6 (2.6–2.6)	4.3 (2.1–6.8)	11.6 (17.4–5.9)	14.4 (22.4–7.7)	100580 (134559–72383)	299310 (416036–202454)	83957 (113594–57482)	147430 (201010–96499)	199127 (285497–124684)	616438 (916818–368480)	167955 (239090–98475)	302938 (442011–176859)
**Northern**	70–79	Men	30% by 2025	3.7 (3.7–3.7)	5.7 (3–8.8)	15 (22.1–8.3)	18.6 (28.2–10.8)	156497 (212380–111762)	442874 (621357–308817)	138886 (184480–101018)	262811 (366239–179892)	323631 (464885–206210)	951783 (1404915–602166)	287751 (403511–186414)	563341 (827918–355372)
**Southern**	35–39	Women	30% by 2025	3.2 (3.2–3.2)	2.5 (0.9–4)	11.8 (21.9–4)	16.5 (26.3–6.2)	10243 (19506–5280)	17467 (27960–7077)	3165 (7092–580)	1839 (5319–0)	14712 (31444–7110)	27400 (46828–9961)	4761 (11047–776)	2891 (9007–0)
**Southern**	35–39	Men	30% by 2025	3.2 (3.2–3.2)	2.7 (1–4.2)	12.4 (22.5–4.4)	17 (26.8–6.8)	14764 (23953–7013)	28197 (44951–14782)	10717 (21116–4067)	6502 (14368–2634)	22517 (39802–9733)	45085 (75836–21306)	16090 (34345–6005)	10464 (23652–3683)

**Southern**	40–44	Women	30% by 2025	2.9 (2.9–2.9)	2.4 (0.9–3.8)	10.9 (20.8–3.3)	15.3 (23.8–5.8)	17696 (28084–11060)	88464 (136789–53263)	4499 (9941–1526)	22945 (37692–12758)	28309 (50416–14732)	149419 (250486–75888)	7195 (17256–2096)	39115 (68319–18453)
**Southern**	40–44	Men	30% by 2025	3.1 (3.1–3.1)	2.7 (1.2–4.1)	12.3 (22.5–5)	17.3 (25.9–7.7)	28533 (45854–15137)	151612 (202965–94337)	20866 (33353–10295)	37392 (55472–21904)	46755 (81098–22062)	263497 (380900–140360)	34440 (59303–15316)	65232 (99513–33268)
**Southern**	45–49	Women	30% by 2025	3 (3–3)	2.6 (1.1–3.9)	10.1 (20.6–4)	14.1 (21.9–6.4)	34293 (52358–21357)	93258 (126540–59217)	12284 (20019–5993)	26417 (37922–17178)	50701 (90644–28039)	151353 (221125–82349)	18748 (33178–8528)	42576 (64440–24531)
**Southern**	45–49	Men	30% by 2025	3.3 (3.3–3.3)	3.2 (1.6–4.5)	11.6 (23.8–6.1)	17.1 (25–9.5)	56728 (81649–40351)	161876 (218085–119366)	42928 (75255–25100)	43247 (57312–28431)	88164 (144571–55470)	271442 (384641–184774)	67592 (131277–35830)	73213 (102820–43299)
**Southern**	50–54	Women	30% by 2025	3.1 (3.1–3.1)	3.1 (1.7–4.3)	13.1 (21.6–6.2)	17.3 (23.6–9.2)	56777 (84762–39789)	387015 (485909–280826)	24500 (35418–13180)	83780 (107687–54156)	88970 (141020–53117)	623973 (826370–410104)	37611 (55419–19184)	136267 (181317–79939)
**Southern**	50–54	Men	30% by 2025	3.3 (3.3–3.3)	3.8 (2.1–5.3)	15.4 (24.5–7.8)	20.2 (27.8–11.4)	108755 (149959–74728)	706139 (892439–495858)	78001 (107479–52076)	131987 (175718–90979)	174072 (256021–106521)	1177272 (1548235–753526)	123653 (183412–76866)	219805 (301737–138580)
**Southern**	55–59	Women	30% by 2025	3.2 (3.2–3.2)	3.9 (2.2–5.8)	12.8 (20.4–7.2)	17.7 (25–10)	66856 (102157–47687)	320128 (423546–240605)	26088 (40120–16726)	66805 (89109–46316)	116266 (187930–74523)	571683 (791953–396535)	45053 (71711–27611)	120332 (165213–77434)
**Southern**	55–59	Men	30% by 2025	3.2 (3.2–3.2)	3.7 (2.1–5.3)	12.3 (19.1–6.7)	16.5 (23.3–9.2)	120414 (160582–80045)	511312 (675324–366689)	66982 (99309–44081)	95030 (131837–63150)	201505 (291238–124692)	908547 (1253206–596694)	114068 (177411–69443)	168822 (241534–106641)
**Southern**	60–64	Women	30% by 2025	2.9 (2.9–2.9)	3.8 (2.2–5.8)	13.1 (20–8)	17.1 (24.4–9.9)	104632 (142719–77845)	615165 (744130–446153)	63916 (86127–47133)	138236 (181689–102660)	195088 (284881–130442)	1214665 (1541523–782473)	121038 (172270–80548)	269751 (377500–181950)
**Southern**	60–64	Men	30% by 2025	3.2 (3.2–3.2)	4.3 (2.5–6.6)	14.8 (22.5–8.9)	19.2 (27.3–11)	185037 (251240–138261)	847776 (1098488–613892)	138271 (186105–105278)	330870 (431860–244933)	354636 (515492–238810)	1715561 (2343966–1102989)	261233 (377332–179779)	667646 (923707–439887)
**Southern**	65–69	Women	30% by 2025	2.8 (2.8–2.8)	4 (2.3–6.2)	10.8 (16.4–5.9)	13.7 (21–8.1)	123849 (163157–97439)	426051 (572121–319395)	96957 (129682–69803)	98053 (134585–74160)	239580 (337027–160578)	863178 (1252363–573591)	185848 (270602–118065)	200310 (295973–130696)
**Southern**	65–69	Men	30% by 2025	2.9 (2.9–2.9)	3.8 (2.2–5.7)	10.1 (15.1–5.9)	12.9 (19.3–7.9)	183247 (222131–139259)	547554 (709315–416452)	158553 (207306–133355)	212788 (270954–156036)	346061 (449946–232172)	1095799 (1529285–747682)	298776 (424804–222379)	424321 (586026–279896)
**Southern**	70–79	Women	30% by 2025	2.4 (2.4–2.4)	3.4 (2–5.3)	9.3 (13.7–5.5)	11.6 (18–7.3)	135032 (177502–102908)	491657 (658512–373158)	111718 (148614–84693)	258004 (354259–197371)	254053 (360357–174834)	975693 (1400604–672521)	209549 (300755–143088)	510983 (759280–357321)
**Southern**	70–79	Men	30% by 2025	2.9 (2.9–2.9)	4.2 (2.4–6.5)	11.3 (16.6–6.8)	14.1 (21.7–8.9)	206364 (265672–154004)	714573 (955207–531392)	186931 (239927–137541)	452095 (599213–332279)	406509 (557898–271860)	1461679 (2090290–997496)	370062 (503664–240877)	923874 (1315209–627858)
**Northern**	35–39	Women	≤5 g/d by 2030	6.3 (6.3–6.3)	5 (1.8–8.1)	21.8 (38.8–7.3)	30.1 (45.7–11.5)	8080 (16140–4783)	11474 (18507–6114)	2267 (4915–667)	1377 (3333–0)	14394 (29711–7692)	21319 (35264–10460)	3932 (8795–1077)	2523 (6334–0)
**Northern**	35–39	Men	≤5 g/d by 2030	7.8 (7.8–7.8)	6.7 (2.9–10.3)	28.3 (47.1–12.3)	38 (53.4–17.5)	13709 (22413–6926)	21741 (33628–10771)	9225 (17123–4220)	4817 (9711–2085)	25290 (43485–11699)	40607 (64513–18129)	16444 (33042–7501)	9143 (18766–3362)
**Northern**	40–44	Women	≤5 g/d by 2030	6.7 (6.7–6.7)	5.5 (2.1–8.6)	23.3 (41.3–8)	31.7 (46.2–13.3)	15861 (27057–8318)	64225 (94375–37697)	4431 (8572–1867)	15579 (26125–8453)	32024 (57533–14441)	132152 (206125–63954)	8285 (18211–3065)	32215 (56712–14794)
**Northern**	40–44	Men	≤5 g/d by 2030	7.6 (7.6–7.6)	6.5 (2.8–10)	27.1 (46.2–11)	36.6 (51.7–17.1)	25553 (41268–15101)	109626 (150212–67434)	17028 (26997–9835)	25715 (40213–16034)	52114 (89884–26093)	233874 (331701–124096)	34990 (58134–16447)	54811 (88583–29883)

Tan M, *et al. bmjnph* 2022; 5:e000408. doi: 10.1136/bmjnph-2021-000408

**Northern**	45–49	Women	≤5 g/d by 2030	6.6 (6.6–6.6)	6.6 (3.5–9.2)	22.8 (43–12.9)	32.4 (44.5–19.3)	28165 (46746–18394)	69957 (91843–50341)	10752 (17476–6228)	17299 (25518–9929)	53129 (95660–30321)	141296 (194059–94999)	20426 (35883–10032)	35083 (53664–18026)
**Northern**	45–49	Men	≤5 g/d by 2030	7.7 (7.7–7.7)	8.6 (4.7–11.8)	29.2 (51.3–16.2)	40 (52.9–24.7)	50800 (73282–36287)	123258 (165090–88236)	38154 (55786–25701)	29523 (40878–20135)	100026 (154284–64055)	257082 (353804–169208)	75748 (118059–46591)	61432 (87901–40438)
**Northern**	50–54	Women	≤5 g/d by 2030	6.5 (6.5–6.5)	7.3 (4–10)	27.7 (42.2–14.1)	35.4 (46.2–20.5)	50359 (82468–35296)	284834 (359452–198171)	21116 (34210–13020)	54245 (75967–37099)	94012 (163206–60762)	553752 (716189–353333)	38616 (67027–21984)	104899 (150919–66146)
**Northern**	50–54	Men	≤5 g/d by 2030	8 (8–8)	10.1 (5.7–14.9)	35.2 (53.2–19.8)	45.3 (59.3–28)	100193 (146779–67309)	544952 (705899–373175)	71258 (105622–52770)	92606 (125316–65280)	194260 (295728–119220)	1084225 (1428978–695192)	139293 (212365–92393)	183946 (253683–122469)
**Northern**	55–59	Women	≤5 g/d by 2030	6.2 (6.2–6.2)	8.7 (5–13.4)	26.9 (41.3–15)	35.1 (48.1–21.8)	64878 (99484–43464)	246943 (323515–168529)	25274 (39181–14680)	48469 (65524–32401)	133614 (212380–82970)	522124 (698552–338565)	51907 (83127–28294)	102256 (141291–65394)
**Northern**	55–59	Men	≤5 g/d by 2030	7.3 (7.3–7.3)	10 (5.7–15.4)	30 (45.5–17)	38.9 (52.9–24.3)	121503 (184293–79289)	427456 (569770–298491)	69491 (102518–45309)	75107 (102235–49861)	252655 (398791–152879)	909788 (1234868–607023)	143894 (220463–86969)	160442 (221369–100379)
**Northern**	60–64	Women	≤5 g/d by 2030	6.5 (6.5–6.5)	9.8 (5.3–15.1)	30.2 (45.1–17.9)	38.3 (52.4–22.4)	102344 (144454–73126)	491548 (654073–337402)	62663 (87281–43394)	105130 (137907–69502)	240569 (358206–156333)	1197771 (1652806–754700)	147878 (216305–94399)	256453 (347245–156166)
**Northern**	60–64	Men	≤5 g/d by 2030	7.6 (7.6–7.6)	11.3 (6.2–17.5)	34 (50–20.5)	42.9 (57.6–25.5)	176396 (253751–128644)	676554 (879679–447215)	133319 (182453–91853)	241966 (318508–159723)	425694 (640306–287619)	1678255 (2235170–1033219)	323224 (454559–202315)	601031 (805403–361102)
**Northern**	65–69	Women	≤5 g/d by 2030	5.6 (5.6–5.6)	9.6 (4.6–15)	23.4 (35.6–12.4)	29.2 (43.5–16.4)	110668 (155279–77020)	320614 (435771–224082)	85769 (123300–60009)	69900 (97295–47827)	273670 (411929–167070)	821428 (1183771–517311)	212435 (326586–127411)	179699 (263365–110373)
**Northern**	65–69	Men	≤5 g/d by 2030	7.1 (7.1–7.1)	11.2 (5.7–17.3)	27.2 (39.7–15.1)	33.4 (48.1–20)	179207 (238461–123078)	445206 (597490–304193)	158138 (213479–107286)	161287 (212926–113755)	454153 (639586–275991)	1168368 (1634607–726751)	401197 (569404–239815)	424380 (583251–271725)
**Northern**	70–79	Women	≤5 g/d by 2030	5 (5–5)	8.2 (4–12.8)	20.8 (30.3–10.8)	25.4 (38.1–14.1)	127468 (173451–87545)	383116 (538374–248946)	106572 (146204–69282)	188590 (260112–119239)	304059 (437242–182922)	944636 (1390481–552079)	256828 (366478–144462)	464166 (670967–264980)
**Northern**	70–79	Men	≤5 g/d by 2030	7 (7–7)	10.8 (5.6–16.6)	26.5 (37.6–15.2)	32.3 (46.5–19.4)	200620 (274906–138146)	571694 (802246–388946)	178229 (238807–124872)	339319 (472879–227518)	496366 (707367–310363)	1454289 (2099483–917462)	441280 (614293–280580)	861332 (1237421–542600)
**Southern**	35–39	Women	≤5 g/d by 2030	6 (6–6)	4.8 (1.8–7.6)	21.1 (37.3–7.4)	28.9 (43.8–11.5)	12192 (24072–6052)	21447 (34897–7855)	3792 (8696–666)	2262 (6644–0)	20722 (44596–9370)	39057 (66590–14189)	6594 (15682–1025)	4114 (12770–0)
**Southern**	35–39	Men	≤5 g/d by 2030	6.1 (6.1–6.1)	5 (2–7.8)	22.2 (38.2–8.2)	29.7 (44.6–12.5)	17960 (29629–8182)	34874 (56004–17667)	12910 (26261–4907)	8009 (17893–3089)	31788 (56508–12432)	64336 (107483–29171)	22849 (49048–8197)	14853 (33754–4740)
**Southern**	40–44	Women	≤5 g/d by 2030	5.6 (5.6–5.6)	4.5 (1.7–7.1)	19.7 (35.6–6.2)	26.8 (40.1–10.7)	21030 (34362–12943)	106999 (168587–60824)	5307 (12092–1764)	28046 (46320–14685)	40132 (72434–17006)	214659 (360858–101519)	10267 (24827–2690)	56105 (98510–24931)
**Southern**	40–44	Men	≤5 g/d by 2030	5.9 (5.9–5.9)	5.1 (2.2–7.7)	21.9 (38.3–9.2)	30.2 (43.3–14.1)	33934 (56062–17772)	184103 (250589–109985)	24959 (40969–11912)	45673 (67995–25186)	66757 (117020–28211)	380606 (545181–191974)	48609 (85637–20124)	94115 (143504–46317)
**Southern**	45–49	Women	≤5 g/d by 2030	5.7 (5.7–5.7)	4.9 (2.1–7.4)	18.2 (35.3–7.4)	25 (37.3–11.8)	39465 (62964–23944)	110091 (152865–66205)	14388 (23911–6821)	31178 (45452–19641)	69367 (128942–34051)	212592 (314460–109125)	25259 (46811–11594)	59773 (91565–32505)
**Southern**	45–49	Men	≤5 g/d by 2030	6.2 (6.2–6.2)	6 (3.1–8.5)	20.8 (40.1–11.3)	29.8 (41.9–17.1)	66467 (98870–45417)	192864 (263734–139418)	50221 (90799–28564)	51897 (69358–32397)	122742 (205480–70816)	385468 (546119–255140)	94509 (186475–47947)	103768 (145802–59884)

**Southern**	50–54	Women	≤5 g/d by 2030	5.9 (5.9–5.9)	5.9 (3.2–8.2)	23.2 (36.8–11.5)	30.1 (39.8–16.6)	67813 (103412–45331)	468442 (595347–328093)	28826 (42455–15384)	101941 (131728–63515)	125612 (200080–71468)	883454 (1171058–562141)	52932 (78277–26443)	192653 (257245–110052)
**Southern**	50–54	Men	≤5 g/d by 2030	6.2 (6.2–6.2)	7.1 (4–10.1)	27 (41.1–14.2)	34.8 (46–20.4)	131872 (183778–86683)	861885 (1095151–588274)	94364 (131747–61526)	161024 (215295–108436)	245652 (362293–142625)	1671135 (2181913–1050057)	173114 (259723–104145)	311752 (425297–192834)
**Southern**	55–59	Women	≤5 g/d by 2030	6 (6–6)	7.4 (4.2–10.9)	22.9 (35–13.2)	30.7 (41.9–18.1)	84722 (130476–58078)	406749 (541031–298256)	32842 (50931–20773)	85125 (113837–57645)	171230 (276967–106354)	846194 (1159478–579298)	66284 (106084–39912)	177982 (242588–113581)
**Southern**	55–59	Men	≤5 g/d by 2030	6 (6–6)	7 (3.9–10)	21.9 (33–12.3)	28.9 (39.3–16.8)	149951 (204497–96984)	649076 (862881–452345)	83891 (126233–53589)	120776 (168334–78622)	296947 (430346–177118)	1344870 (1840986–867579)	168248 (262492–99867)	249878 (356322–157300)
**Southern**	60–64	Women	≤5 g/d by 2030	5.6 (5.6–5.6)	7.2 (4.1–10.9)	23.4 (34.4–14.7)	29.9 (41–17.8)	128981 (178635–93052)	768153 (938966–538614)	78695 (107722–56481)	172998 (229031–124716)	289570 (425764–188590)	1815316 (2295111–1146380)	180627 (257373–116341)	402391 (560930–266681)
**Southern**	60–64	Men	≤5 g/d by 2030	6.1 (6.1–6.1)	8.2 (4.6–12.4)	26 (38.3–16.2)	33.1 (45.3–19.8)	229371 (316259–166399)	1065080 (1388673–747131)	171141 (233926–126340)	415634 (545882–297963)	529081 (768315–348146)	2562566 (3458265–1626432)	389455 (562939–261719)	997075 (1361227–648645)
**Southern**	65–69	Women	≤5 g/d by 2030	5.2 (5.2–5.2)	7.6 (4.3–11.7)	19.3 (28.7–10.8)	24.2 (36–14.8)	149889 (200416–112186)	521337 (710671–377192)	115949 (159642–81213)	119415 (167028–86724)	356992 (507022–228025)	1295785 (1878633–840145)	277330 (407137–169197)	301271 (443462–191521)
**Southern**	65–69	Men	≤5 g/d by 2030	5.6 (5.6–5.6)	7.2 (4.1–10.8)	18.1 (26.6–10.9)	22.9 (33.2–14.5)	218942 (271532–162096)	667153 (879142–491802)	190835 (254140–153962)	259104 (335997–184220)	514390 (675845–329840)	1644012 (2298618–1093306)	442900 (638882–317686)	636021 (880502–409837)
**Southern**	70–79	Women	≤5 g/d by 2030	4.5 (4.5–4.5)	6.4 (3.7–9.9)	16.9 (24.3–10.2)	20.8 (31.3–13.3)	167634 (226300–122920)	622972 (848577–457654)	138968 (189144–101429)	326695 (457267–242211)	384909 (551855–256532)	1489939 (2142835–1004130)	317473 (460623–208921)	780313 (1161049–534537)
**Southern**	70–79	Men	≤5 g/d by 2030	5.5 (5.5–5.5)	8 (4.5–12.2)	20.4 (29.1–12.4)	25 (37.1–16.1)	260560 (341663–187315)	913198 (1235848–660657)	235886 (308606–167439)	577404 (775267–414296)	620118 (855053–403590)	2239609 (3178986–1507295)	565252 (772088–356766)	1415544 (1997561–949531)

Supplementary Table 4.2. Reduction in salt intake, systolic blood pressure, and CVD risk, events, deaths, and premature deaths, with effect size of salt reduction based on a population study (more prolonged effect)

All results are reported in median (95% uncertainty interval). The changes reported for salt intake and SBP are absolute reductions; those reported for disease risk are relative reductions. Abbreviations: IHD = ischaemic heart disease, SBP = systolic blood pressure.

Region	Age groups (years)	Sex	Salt reduc-tions	↓ salt intake (g/d)	↓ SBP (mm Hg)	↓ IHD risk (%)	↓ stroke risk (%)	↓ IHD events by 2030 (n)	↓ stroke events by 2030 (n)	↓ IHD deaths by 2030 (n)	↓ stroke deaths by 2030 (n)	↓ IHD events by 2040 (n)	↓ stroke events by 2040 (n)	↓ IHD deaths by 2040 (n)	↓ stroke deaths by 2040 (n)
**Northern**	35–39	Women	1 g/d in a year	1 (1–1)	1.9 (1.4–2.4)	9.2 (16.7–5.6)	13.1 (16.1–9.6)	6839 (11207–3879)	9413 (13713–5787)	1798 (3695–554)	911 (2528–0)	9559 (16551–5270)	13948 (20525–8444)	2547 (5500–743)	1372 (3791–0)
**Northern**	35–39	Men	1 g/d in a year	1 (1–1)	1.9 (1.5–2.4)	9.2 (16.6–5.6)	13.1 (15.9–9.9)	8982 (14983–5340)	14295 (19961–9528)	6723 (11163–3711)	3246 (5841–1377)	12649 (22554–7327)	21048 (29786–13723)	9463 (16861–4962)	4788 (8653–2062)
**Northern**	40–44	Women	1 g/d in a year	1 (1–1)	1.9 (1.5–2.4)	9.5 (16.7–5.6)	13 (16–9.9)	12577 (18935–7996)	48219 (59787–38073)	3463 (6380–1416)	12341 (17474–8150)	18941 (30242–11184)	76722 (97096–58672)	5226 (9967–2091)	19677 (28322–12724)
**Northern**	40–44	Men	1 g/d in a year	1 (1–1)	1.9 (1.5–2.4)	9.5 (16.6–5.6)	13 (16–9.9)	17991 (27142–12598)	74089 (89367–60486)	12553 (18992–7942)	17913 (24389–12565)	26982 (44571–17571)	117370 (145184–93160)	18803 (30599–11201)	28442 (39365–19747)
**Northern**	45–49	Women	1 g/d in a year	1 (1–1)	1.9 (1.5–2.3)	8.1 (12.7–5.1)	11.1 (13.3–8.9)	21809 (29780–16004)	49431 (59804–39923)	8138 (12813–4817)	12746 (17776–8407)	30818 (45156–21208)	73766 (90995–58294)	11404 (19005–6616)	19032 (26909–12545)
**Northern**	45–49	Men	1 g/d in a year	1 (1–1)	1.9 (1.6–2.3)	8.1 (12.6–5.2)	11.1 (13.2–9)	33669 (45632–25585)	76326 (90896–63867)	25089 (33889–18169)	18436 (24646–12825)	47687 (68793–33940)	114067 (138092–93058)	35414 (50411–24620)	27456 (37000–18767)
**Northern**	50–54	Women	1 g/d in a year	1 (1–1)	1.9 (1.6–2.3)	8.2 (13.2–5.2)	11.1 (13.1–8.8)	34535 (46936–25041)	181264 (206407–157787)	14151 (20262–9431)	35110 (44166–27115)	48155 (69807–33418)	264954 (308497–224231)	19682 (29739–12737)	51147 (65216–39033)
**Northern**	50–54	Men	1 g/d in a year	1 (1–1)	1.9 (1.6–2.2)	8.2 (13.1–5.3)	11.1 (13–9)	55912 (74474–43792)	287927 (322557–252905)	39803 (53315–30099)	49786 (60452–39380)	77825 (110354–57969)	419700 (480061–359570)	55473 (78548–40060)	72569 (89123–56883)
**Northern**	55–59	Women	1 g/d in a year	1 (1–1)	1.9 (1.6–2.3)	6.9 (9.6–4.9)	9 (10.6–7.4)	34895 (44514–27412)	125912 (142591–109250)	13310 (18351–9500)	24400 (30192–18805)	52116 (69675–39628)	197231 (227945–166689)	19789 (28556–13773)	38153 (48164–28935)
**Northern**	55–59	Men	1 g/d in a year	1 (1–1)	1.9 (1.6–2.2)	6.9 (9.6–4.9)	9 (10.6–7.4)	59551 (74242–47522)	199210 (226481–175070)	34327 (44445–27122)	34317 (42015–27282)	89242 (116289–67955)	311857 (361864–266484)	51285 (69979–39016)	53590 (66424–42320)
**Northern**	60–64	Women	1 g/d in a year	1 (1–1)	1.9 (1.6–2.3)	6.8 (9.3–4.9)	9 (10.7–7.3)	55347 (67793–46288)	253200 (282750–224573)	33217 (41642–26599)	54172 (63165–45396)	86670 (112568–68907)	419859 (483854–359329)	51869 (68518–39861)	89809 (106604–73898)
**Northern**	60–64	Men	1 g/d in a year	1 (1–1)	1.9 (1.6–2.3)	6.8 (9.3–4.9)	9 (10.7–7.3)	87732 (105674–74406)	316765 (351757–285988)	65963 (79068–55636)	115226 (130079–100248)	137172 (174930–109477)	525916 (601399–456211)	103394 (130393–82305)	191323 (221401–160589)
**Northern**	65–69	Women	1 g/d in a year	1 (1–1)	1.9 (1.5–2.3)	5.3 (6.7–4.2)	6.8 (8.5–5.4)	64862 (74117–56964)	176808 (198512–157625)	49542 (57216–43236)	37959 (45144–31872)	102142 (121317–85939)	294422 (342841–251040)	77940 (92949–65958)	62895 (77522–51392)
**Northern**	65–69	Men	1 g/d in a year	1 (1–1)	1.9 (1.6–2.3)	5.3 (6.6–4.3)	6.8 (8.3–5.5)	93854 (105799–83111)	221145 (245659–199127)	83319 (94032–73537)	80527 (91660–70716)	147441 (173462–125591)	367561 (424486–317173)	130867 (153326–112527)	133937 (158451–114167)

Tan M, *et al. bmjnph* 2022; 5:e000408. doi: 10.1136/bmjnph-2021-000408

**Northern**	70–79	Women	1 g/d in a year	1 (1–1)	1.9 (1.6–2.3)	5.4 (6.6–4.2)	6.8 (8.3–5.5)	101083 (113515–90410)	289622 (320874–265530)	84756 (95342–74972)	140252 (156532–125567)	146018 (169670–125841)	439764 (503235–385705)	122389 (142506–105043)	213303 (245751–184094)
**Northern**	70–79	Men	1 g/d in a year	1 (1–1)	1.9 (1.6–2.3)	5.3 (6.6–4.2)	6.8 (8.3–5.5)	134525 (149994–120898)	357605 (392686–327175)	120977 (134456–108778)	211507 (234222–190268)	194062 (223515–167476)	541960 (618474–476904)	174355 (201719–150348)	320202 (369908–275764)
**Southern**	35–39	Women	1 g/d in a year	1 (1–1)	2 (1.4–2.4)	9.5 (16.7–5.6)	13 (16–9.9)	18785 (30533–11409)	89623 (112052–69984)	5123 (10419–1513)	24413 (35761–15276)	28121 (49076–16289)	142168 (182251–107390)	7708 (16263–2239)	38799 (57675–23774)
**Southern**	35–39	Men	1 g/d in a year	1 (1–1)	1.9 (1.5–2.4)	9.5 (16.6–5.6)	13 (15.9–10)	28207 (42788–18229)	142049 (172694–115285)	19601 (32462–11658)	36610 (51550–24713)	42653 (69672–25961)	225389 (280534–177980)	29336 (52215–17128)	58365 (82810–39094)
**Southern**	40–44	Women	1 g/d in a year	1 (1–1)	1.9 (1.5–2.4)	8.1 (12.9–5)	11.1 (13.5–8.6)	33203 (48551–22592)	92654 (113030–73330)	12393 (19989–6050)	25421 (37050–16452)	46828 (72420–30214)	138362 (171636–106782)	17385 (29444–8593)	38036 (55961–24877)
**Southern**	40–44	Men	1 g/d in a year	1 (1–1)	1.9 (1.5–2.3)	8.1 (12.6–5.1)	11.1 (13.3–8.8)	52207 (73216–37988)	145655 (174759–120869)	39101 (54269–26978)	38094 (52035–25501)	73476 (111062–51568)	217403 (266297–175851)	55298 (81315–36468)	56922 (77698–37853)
**Southern**	45–49	Women	1 g/d in a year	1 (1–1)	1.9 (1.5–2.3)	8.1 (13.3–5.2)	11.1 (13.2–8.7)	52085 (74341–37162)	339194 (389644–288351)	21223 (30912–13057)	70897 (88466–53547)	72561 (109698–48814)	495574 (578181–407780)	29536 (45483–17538)	103391 (131091–77117)
**Southern**	45–49	Men	1 g/d in a year	1 (1–1)	1.9 (1.6–2.3)	8.2 (13.1–5.2)	11.1 (13.1–8.9)	86687 (117098–64633)	556537 (631340–486845)	61536 (84529–45416)	103566 (125423–82086)	120455 (170721–86588)	813148 (940353–694073)	85842 (126025–61492)	150765 (184945–118001)
**Southern**	50–54	Women	1 g/d in a year	1 (1–1)	1.9 (1.6–2.2)	6.9 (9.6–4.9)	9 (10.6–7.4)	52547 (68147–40620)	234885 (272827–200339)	20162 (28808–13712)	49441 (62991–37423)	78528 (105753–58768)	367686 (434646–306498)	30120 (44424–19850)	77446 (99960–57538)
**Southern**	50–54	Men	1 g/d in a year	1 (1–1)	1.9 (1.6–2.3)	6.9 (9.6–4.9)	9 (10.6–7.3)	92974 (118391–74553)	385829 (440297–335525)	53986 (71266–40833)	71367 (88189–56280)	139052 (186491–106044)	603297 (702677–512459)	80346 (110363–58637)	111654 (140023–86475)
**Southern**	55–59	Women	1 g/d in a year	1 (1–1)	1.9 (1.6–2.2)	6.8 (9.4–4.9)	9 (10.6–7.5)	84439 (105746–68222)	473269 (525854–418814)	50371 (63138–39410)	109345 (129051–90853)	131832 (174946–102726)	787329 (895137–675662)	78537 (104889–59049)	181489 (218324–147627)
**Southern**	55–59	Men	1 g/d in a year	1 (1–1)	1.9 (1.6–2.2)	6.8 (9.3–4.9)	9 (10.7–7.5)	138217 (165342–114915)	612252 (682043–548786)	103847 (126914–84525)	240535 (273995–209879)	215713 (273345–171135)	1016782 (1166006–880397)	161424 (208159–127010)	398627 (466294–336912)
**Southern**	60–64	Women	1 g/d in a year	1 (1–1)	1.9 (1.6–2.3)	5.3 (6.5–4.3)	6.9 (8.2–5.6)	97716 (114030–84829)	332513 (369882–296217)	75795 (88500–63852)	76214 (88523–64018)	153904 (184581–129421)	553497 (638809–475945)	119435 (143048–98106)	126776 (151055–104047)
**Southern**	60–64	Men	1 g/d in a year	1 (1–1)	1.9 (1.6–2.2)	5.4 (6.5–4.4)	6.9 (8.3–5.6)	146817 (166435–127768)	428467 (479827–383147)	131025 (149067–114230)	168297 (192838–147294)	230840 (269158–195551)	714633 (824970–614904)	205803 (243334–173100)	279452 (332088–238878)
**Southern**	65–69	Women	1 g/d in a year	1 (1–1)	1.9 (1.6–2.3)	5.3 (6.5–4.3)	6.8 (8.2–5.5)	153637 (173816–136037)	543373 (595350–494206)	128426 (146814–112701)	283749 (317381–254299)	222722 (258798–191954)	826505 (938056–723990)	185960 (220024–159485)	431146 (497550–375194)
**Southern**	65–69	Men	1 g/d in a year	1 (1–1)	1.9 (1.6–2.2)	5.4 (6.5–4.3)	6.8 (8.2–5.6)	211856 (237000–189195)	691513 (758343–629575)	190276 (213997–168810)	440283 (487686–396975)	305706 (354316–264743)	1052160 (1191347–923308)	275032 (318953–237144)	669294 (764605–581646)
**Southern**	70–79	Women	1 g/d in a year	1 (1–1)	1.9 (1.5–2.3)	7.1 (14–4.6)	9.9 (15–6)	824163 (1004739–687246)	2681230 (3023801–2372695)	597128 (725059–495520)	848850 (990282–719974)	1227480 (1569453–978700)	4200146 (4874333–3588687)	889900 (1128576–711861)	1327695 (1592604–1092919)
**Southern**	70–79	Men	1 g/d in a year	1 (1–1)	1.9 (1.5–2.3)	7.1 (14–4.6)	9.9 (15–6)	1273620 (1577449–1040619)	5112562 (5783345–4488048)	926083 (1145525–748403)	1746864 (2049447–1477256)	1896312 (2452615–1497026)	8020178 (9308829–6822400)	1380377 (1779499–1083500)	2734993 (3287641–2252877)

**Northern**	35–39	Women	30% by 2025	3.4 (3.4–3.4)	6.5 (4.8–8)	27.6 (45.8–17.6)	37.6 (44.5–28.8)	11926 (20445–6561)	17289 (25345–10476)	3171 (6764–917)	1687 (4652–0)	20235 (35926–10908)	30274 (44541–18214)	5379 (11852–1480)	2982 (8215–0)
**Northern**	35–39	Men	30% by 2025	4.1 (4.1–4.1)	8 (6.1–9.7)	32.7 (52.7–21.2)	43.9 (51.1–34.9)	17926 (31521–10257)	29648 (41749–19450)	13441 (23142–6993)	6746 (12174–2896)	30770 (56096–17458)	52533 (74420–34313)	23235 (41539–11780)	11943 (21625–5117)
**Northern**	40–44	Women	30% by 2025	3.5 (3.5–3.5)	6.9 (5.1–8.4)	29.7 (47.5–18.3)	38.9 (46–30.8)	22141 (34891–13098)	88286 (110591–68412)	6077 (11511–2448)	22660 (32223–14765)	42058 (68282–23691)	173283 (219152–131267)	11492 (22249–4520)	44424 (63921–28635)
**Northern**	40–44	Men	30% by 2025	4 (4–4)	7.8 (5.9–9.5)	33.2 (51.9–20.6)	42.9 (50.3–34.3)	34162 (54237–22215)	146546 (178872–117446)	23703 (37649–14145)	35434 (48761–24651)	65524 (109654–40400)	289908 (357633–229638)	45717 (74858–26057)	70258 (96890–48401)
**Northern**	45–49	Women	30% by 2025	3.5 (3.5–3.5)	6.8 (5.4–8.1)	25.6 (37.7–16.8)	33.8 (39.3–27.7)	35553 (51581–24344)	84599 (103969–67249)	13139 (21704–7605)	21831 (30726–14392)	63783 (97137–41564)	158611 (197519–123915)	23621 (40819–13105)	40932 (57998–26815)
**Northern**	45–49	Men	30% by 2025	4.1 (4.1–4.1)	7.9 (6.4–9.3)	29.2 (42.3–19.4)	38.2 (43.9–31.9)	60081 (86365–42204)	143462 (172789–117466)	44576 (63220–30490)	34563 (46398–23697)	110077 (165306–73151)	272688 (331228–219289)	81566 (120506–53366)	65679 (89042–44493)
**Northern**	50–54	Women	30% by 2025	3.5 (3.5–3.5)	6.7 (5.4–7.8)	25.5 (38.6–16.8)	33.5 (38.5–27.2)	59146 (85233–40499)	324902 (375692–275624)	24173 (36358–15550)	62757 (79860–47967)	101098 (152813–66755)	576554 (675726–480228)	41417 (65032–25875)	111247 (142247–84371)
**Northern**	50–54	Men	30% by 2025	4.2 (4.2–4.2)	8.2 (6.7–9.5)	30.2 (44.7–20.4)	39.1 (44.5–32.7)	107753 (151722–78427)	582278 (661159–499546)	76770 (108616–54495)	100475 (122942–78932)	189106 (276584–131369)	1049359 (1203924–881525)	134411 (196849–91864)	181174 (223205–141256)
**Northern**	55–59	Women	30% by 2025	3.3 (3.3–3.3)	6.4 (5.3–7.4)	20.9 (28.3–15.2)	26.7 (31–22.3)	63493 (84764–48116)	239669 (275724–203009)	24098 (34753–16741)	46378 (58236–35223)	116573 (159333–85270)	451440 (525518–377930)	44255 (65222–29997)	87292 (111070–65858)
**Northern**	55–59	Men	30% by 2025	3.8 (3.8–3.8)	7.4 (6.2–8.6)	23.9 (32.1–17.4)	30.4 (35–25.6)	120210 (156314–90986)	420438 (485191–359888)	69126 (94029–52022)	72241 (89397–57055)	223791 (298619–162326)	800966 (935725–675120)	128480 (178398–93609)	137673 (171384–108029)
**Northern**	60–64	Women	30% by 2025	3.5 (3.5–3.5)	6.6 (5.5–7.8)	21.5 (28.5–16)	27.8 (32.3–23.1)	94166 (121093–75039)	451940 (516470–388910)	56372 (73843–43384)	96719 (114338–79842)	193748 (260697–149160)	970485 (1129761–815235)	116005 (157401–85726)	207488 (248127–167551)
**Northern**	60–64	Men	30% by 2025	4 (4–4)	7.7 (6.4–9.1)	24.5 (32.3–18.3)	31.5 (36.4–26.4)	163116 (206899–129732)	621161 (704747–541920)	122852 (154495–97723)	225836 (260340–190290)	342598 (450868–260412)	1355668 (1566231–1153895)	257809 (335252–196249)	493300 (574971–407985)
**Northern**	65–69	Women	30% by 2025	3 (3–3)	5.7 (4.6–6.9)	14.9 (18.5–11.9)	18.9 (23–15.1)	98811 (116639–83794)	281642 (325092–242419)	75416 (89462–64349)	60233 (73479–49602)	203467 (249385–165256)	608228 (723812–503432)	155401 (190601–126896)	129725 (162851–103906)
**Northern**	65–69	Men	30% by 2025	3.8 (3.8–3.8)	7.3 (5.9–8.6)	18.7 (22.6–15.2)	23.5 (27.8–19.3)	163941 (192474–139505)	407269 (467845–352492)	145512 (170173–125041)	148323 (174256–126904)	352022 (425399–288513)	912237 (1068824–766834)	312720 (377276–258953)	331630 (398753–275333)
**Northern**	70–79	Women	30% by 2025	2.5 (2.5–2.5)	4.8 (3.9–5.7)	12.8 (15.7–10.2)	16.2 (19.4–13.2)	136337 (157100–118509)	405596 (460044–360168)	114406 (131683–98926)	196567 (224079–170924)	244478 (292406–203314)	762280 (888416–650700)	204768 (244999–169831)	369851 (435950–309524)
**Northern**	70–79	Men	30% by 2025	3.5 (3.5–3.5)	6.7 (5.5–8)	17.5 (21.3–13.9)	21.9 (26.2–17.8)	216010 (248967–185590)	603446 (687350–531023)	194031 (224439–166935)	356476 (410635–306904)	410557 (489917–338951)	1197504 (1405730–1016195)	369645 (442129–304974)	708405 (837408–590173)
**Southern**	35–39	Women	30% by 2025	2.9 (2.9–2.9)	5.7 (4.3–7)	25.5 (41.6–15.6)	33.6 (40.2–26.4)	29542 (50089–17233)	146910 (185476–112120)	8043 (16815–2360)	40089 (59125–24738)	54706 (97492–30223)	282485 (363609–211839)	14924 (31991–4240)	77094 (114864–47069)
**Southern**	35–39	Men	30% by 2025	3.1 (3.1–3.1)	6 (4.6–7.3)	26.7 (43.1–16.3)	35.1 (41.6–27.9)	46052 (73235–28286)	240644 (295871–192282)	31749 (55226–18674)	62164 (88219–41807)	85848 (143841–50211)	465798 (580752–362071)	59128 (107744–32728)	120656 (171236–79774)

**Southern**	40–44	Women	30% by 2025	3 (3–3)	5.8 (4.4–7.1)	22.4 (33.8–14.3)	29.8 (35.3–23.5)	49654 (76210–32115)	145112 (178915–112870)	18435 (30811–9099)	39847 (58578–25927)	87215 (139688–53887)	267455 (334223–202973)	32434 (56612–15266)	73335 (109383–46993)
**Southern**	40–44	Men	30% by 2025	3.3 (3.3–3.3)	6.4 (5–7.6)	24.2 (35.8–15.8)	32.1 (37.5–26.1)	81862 (122840–57413)	240620 (292848–195656)	61536 (89788–40641)	62876 (86227–42159)	145840 (228870–96094)	446579 (552299–356026)	109419 (168065–69016)	117160 (160989–76935)
**Southern**	45–49	Women	30% by 2025	3.1 (3.1–3.1)	6.1 (4.8–7.2)	23.3 (36–15.2)	30.7 (35.8–24.8)	83708 (125538–56225)	570034 (660325–470725)	34065 (52102–20187)	119059 (150311–88873)	142121 (223190–92140)	1002381 (1180071–815320)	57543 (92336–33145)	209263 (267124–154009)
**Southern**	45–49	Men	30% by 2025	3.3 (3.3–3.3)	6.4 (5.2–7.4)	24.3 (37–16.1)	32 (36.9–26.2)	143087 (202176–102003)	964384 (1105775–824374)	102039 (148413–72468)	178858 (218867–140244)	244886 (359889–166756)	1702316 (1984135–1429120)	174318 (265538–116557)	315306 (389681–245050)
**Southern**	50–54	Women	30% by 2025	3.2 (3.2–3.2)	6.2 (5–7.1)	20.3 (27.5–14.6)	26 (30–21.6)	93344 (125368–69787)	436006 (513686–364460)	35776 (52655–23481)	91807 (118267–68425)	170085 (235637–123937)	819446 (973857–672936)	64853 (97908–41898)	172412 (223475–127816)
**Southern**	50–54	Men	30% by 2025	3.2 (3.2–3.2)	6.1 (5–7.1)	20.2 (27.4–14.7)	25.9 (29.9–21.3)	164874 (220084–124849)	713108 (827501–607065)	95209 (130575–69236)	131997 (164857–102292)	300131 (414530–219898)	1337406 (1570159–1120434)	173326 (245915–122361)	247678 (312800–189513)
**Southern**	55–59	Women	30% by 2025	2.9 (2.9–2.9)	5.7 (4.7–6.6)	18.7 (25.1–13.7)	24.2 (28.2–20.4)	130412 (170489–102292)	766959 (866253–664959)	77631 (101705–58878)	176859 (211755–145123)	261633 (357725–195626)	1610615 (1855072–1361231)	155827 (213294–113784)	370822 (452282–297808)
**Southern**	55–59	Men	30% by 2025	3.2 (3.2–3.2)	6.3 (5.2–7.3)	20.4 (27.2–15.1)	26.3 (30.6–22.2)	225682 (283138–179932)	1053240 (1196689–917334)	168971 (216118–133763)	412992 (478484–351609)	458734 (599683–349353)	2233912 (2593078–1906357)	344934 (457551–260038)	877741 (1035086–726383)
**Southern**	60–64	Women	30% by 2025	2.8 (2.8–2.8)	5.3 (4.4–6.3)	14.1 (17–11.6)	17.9 (21.2–14.8)	143344 (171219–121638)	508691 (580556–441086)	111340 (132078–92078)	116499 (137828–96418)	292113 (357643–238738)	1084060 (1271843–909463)	226576 (276981–182446)	248536 (300277–200275)
**Southern**	60–64	Men	30% by 2025	2.9 (2.9–2.9)	5.7 (4.7–6.6)	15 (17.9–12.3)	18.9 (22.4–15.7)	222854 (258407–189734)	681328 (779358–591812)	198563 (232748–168471)	266707 (313563–230003)	458300 (546642–379524)	1469174 (1727469–1235758)	408789 (492079–333261)	574563 (693442–480734)
**Southern**	65–69	Women	30% by 2025	2.4 (2.4–2.4)	4.7 (3.9–5.5)	12.5 (15.2–10.1)	15.9 (18.9–13)	205591 (236709–178491)	752480 (843514–665299)	171632 (201145–148168)	392240 (447794–343280)	367431 (439396–307395)	1410065 (1631062–1203483)	306892 (373145–256342)	736091 (863376–620344)
**Southern**	65–69	Men	30% by 2025	2.8 (2.8–2.8)	5.4 (4.5–6.3)	14.4 (17.3–11.6)	18.1 (21.5–14.9)	303232 (349467–263833)	1034357 (1161965–913948)	272786 (315012–235545)	658293 (746371–576570)	556668 (660517–466347)	1986124 (2312154–1693536)	501721 (596395–418437)	1268182 (1479073–1070349)
**Southern**	70–79	Women	30% by 2025	3.5 (2.5–4.2)	7 (4.6–9.1)	23.7 (43.6–12.4)	31.9 (46.9–15.6)	1404773 (1800244–1108876)	4848171 (5592628–4155498)	1006861 (1281839–797766)	1488925 (1782497–1224043)	2709886 (3588423–2058498)	9662018 (11348161–8077730)	1955921 (2564982–1494284)	2994003 (3643658–2407446)
**Southern**	70–79	Men	30% by 2025	3.1 (2.4–3.3)	5.9 (4.4–7.2)	20.6 (37.5–11.9)	27.8 (39.9–15.1)	1964341 (2539736–1544591)	8333425 (9605954–7121201)	1410293 (1818212–1102336)	2765653 (3313318–2282146)	3694982 (4935850–2804971)	16256701 (19135511–13563200)	2668574 (3549904–2015074)	5435774 (6630946–4371096)
**Northern**	35–39	Women	≤5 g/d by 2030	6.3 (6.3–6.3)	12.3 (9.1–15.2)	45.7 (68.6–30.6)	58.9 (67.1–47.4)	14884 (25237–8156)	21408 (31120–13060)	3954 (8385–1138)	2076 (5762–0)	28667 (49120–15541)	41794 (60805–25266)	7582 (16299–2114)	4059 (11253–0)
**Northern**	35–39	Men	≤5 g/d by 2030	7.8 (7.8–7.8)	15.2 (11.5–18.4)	52.7 (75.7–36.2)	66.4 (74.1–55.5)	22287 (38157–12777)	36489 (50999–24134)	16727 (28155–8726)	8296 (14903–3526)	43156 (74442–24480)	71317 (99574–47028)	32379 (54705–16700)	16215 (29118–6890)
**Northern**	40–44	Women	≤5 g/d by 2030	6.7 (6.7–6.7)	13 (9.7–15.9)	48.7 (70.4–31.8)	60.5 (68.8–50.1)	27266 (42345–16063)	108269 (134635–84136)	7491 (14137–3022)	27725 (39371–18162)	59704 (94075–34192)	239885 (299515–185944)	16301 (31259–6461)	61579 (87277–40120)

**Northern**	40–44	Men	≤5 g/d by 2030	7.6 (7.6–7.6)	14.8 (11.2–18)	53.3 (74.9–35.3)	65.3 (73.3–54.8)	41986 (65087–27396)	178670 (215838–144239)	29198 (45466–17446)	43165 (58909–29995)	92395 (144676–58275)	397346 (480184–318581)	64335 (100372–37484)	95972 (131002–66639)
**Northern**	45–49	Women	≤5 g/d by 2030	6.6 (6.6–6.6)	12.8 (10.2–15.2)	42.7 (59–29.4)	54.1 (61–45.8)	43095 (62190–29257)	102670 (125487–81659)	15899 (26278–9154)	26494 (37180–17457)	90248 (135159–59083)	221240 (272567–174537)	33481 (56634–18638)	57018 (80582–37571)
**Northern**	45–49	Men	≤5 g/d by 2030	7.7 (7.7–7.7)	15 (12.1–17.5)	47.9 (64.7–33.5)	59.7 (66.4–51.6)	72936 (104171–50911)	173366 (208171–142563)	54135 (76659–36959)	41759 (55972–28776)	155512 (226411–104063)	376080 (452522–307152)	114898 (165620–75825)	90503 (121630–61597)
**Northern**	50–54	Women	≤5 g/d by 2030	6.5 (6.5–6.5)	12.7 (10.2–14.8)	42.6 (60.3–29.4)	53.7 (60.1–45.1)	72369 (103935–49426)	397828 (457815–338093)	29577 (44523–18952)	76793 (97623–58822)	143027 (210202–94789)	801657 (927002–674115)	58499 (89785–36740)	154532 (196989–117851)
**Northern**	50–54	Men	≤5 g/d by 2030	8 (8–8)	15.4 (12.7–17.9)	49.3 (67.4–35)	60.8 (67.1–52.7)	132173 (182736–95774)	709256 (800416–610970)	94139 (132397–66715)	122311 (149550–96061)	264750 (370397–186485)	1436241 (1623251–1231973)	188357 (267374–130422)	247680 (303092–194677)
**Northern**	55–59	Women	≤5 g/d by 2030	6.2 (6.2–6.2)	12 (10–14.1)	35.8 (46.7–26.7)	44.4 (50.3–38)	81062 (108298–61194)	305877 (350993–259408)	30801 (44394–21272)	59245 (74084–44995)	171839 (232612–126278)	658303 (758673–554266)	65204 (95318–44402)	127283 (160232–96436)
**Northern**	55–59	Men	≤5 g/d by 2030	7.3 (7.3–7.3)	14 (11.7–16.3)	40.4 (51.8–30.4)	49.5 (55.7–42.7)	153617 (198638–115666)	535693 (614404–459620)	88312 (119343–66322)	91967 (113553–72722)	328066 (429665–240545)	1156677 (1332968–988054)	188655 (258217–138651)	198764 (245899–156996)
**Northern**	60–64	Women	≤5 g/d by 2030	6.5 (6.5–6.5)	12.6 (10.3–14.8)	36.7 (47–28)	46 (52.1–39.2)	118658 (152953–93891)	571424 (651504–491501)	70967 (93373–54260)	122286 (144469–100942)	288889 (383145–223128)	1427958 (1642732–1211923)	173215 (232901–128240)	305418 (362812–249278)
**Northern**	60–64	Men	≤5 g/d by 2030	7.6 (7.6–7.6)	14.6 (12–17.2)	41.2 (52.2–31.8)	51.1 (57.5–43.9)	206122 (261301–162914)	783720 (885218–685296)	155253 (195029–122709)	285145 (328061–240592)	508691 (658497–389669)	1977964 (2249781–1708538)	382997 (490324–293596)	719021 (830248–601905)
**Northern**	65–69	Women	≤5 g/d by 2030	5.6 (5.6–5.6)	10.8 (8.6–13)	26.3 (32–21.3)	32.7 (39–26.6)	121364 (144137–101916)	348962 (404723–298260)	92583 (110396–78141)	74536 (91380–61056)	305957 (375178–247242)	913900 (1080786–757642)	233827 (286690–189959)	194923 (243632–156336)
**Northern**	65–69	Men	≤5 g/d by 2030	7.1 (7.1–7.1)	13.7 (11.2–16.3)	32.3 (38.3–26.8)	39.7 (46–33.4)	203037 (239592–171395)	506792 (582871–437236)	180286 (211675–153724)	184658 (216957–157174)	529738 (638233–434362)	1361990 (1582449–1150609)	470451 (563848–389682)	495725 (590869–413355)
**Northern**	70–79	Women	≤5 g/d by 2030	4.7 (4.7–4.7)	9.1 (7.4–10.7)	22.8 (27.5–18.4)	28.4 (33.5–23.4)	165871 (192912–142597)	499668 (570765–438075)	139015 (162001–119110)	242223 (278263–209100)	358711 (431884–295358)	1127021 (1314802–954717)	300506 (361816–245369)	546783 (645132–454458)
**Northern**	70–79	Men	≤5 g/d by 2030	6.6 (6.6–6.6)	12.7 (10.3–15.1)	30.5 (36.4–24.7)	37.3 (43.6–31)	267151 (310372–227415)	752769 (859768–657568)	239997 (280227–204693)	444922 (514047–380487)	607846 (725065–499360)	1769282 (2060011–1504267)	547221 (654125–449237)	1046837 (1228866–873706)
**Southern**	35–39	Women	≤5 g/d by 2030	5.6 (5.6–5.6)	10.8 (8–13.3)	42.6 (63.7–27.3)	53.9 (62.2–43.9)	36382 (61505–21174)	180794 (227450–138427)	9870 (20702–2910)	49370 (72653–30539)	78495 (136157–43650)	398154 (503746–302296)	21412 (45681–6111)	108774 (160668–66897)
**Southern**	35–39	Men	≤5 g/d by 2030	5.9 (5.9–5.9)	11.4 (8.7–13.8)	44.4 (65.5–28.6)	55.8 (63.8–46)	56812 (89526–34670)	295741 (361936–237415)	39161 (67967–22678)	76374 (107890–51304)	122567 (198720–72497)	653831 (804210–516577)	84833 (149624–47261)	169172 (239702–113747)
**Southern**	40–44	Women	≤5 g/d by 2030	5.7 (5.7–5.7)	11 (8.4–13.4)	38.1 (54.1–25.3)	48.8 (56–39.7)	60069 (92402–38565)	176303 (217318–136890)	22272 (37401–10954)	48400 (70946–31473)	123778 (196261–76274)	375688 (466820–287932)	45965 (79521–21612)	103398 (152557–66750)
**Southern**	40–44	Men	≤5 g/d by 2030	6.2 (6.2–6.2)	12.1 (9.4–14.4)	40.8 (56.7–27.8)	51.9 (58.8–43.5)	99042 (149144–69117)	292149 (355096–237557)	74535 (109106–48804)	76331 (104779–51167)	206721 (320471–135800)	626557 (766383–504149)	155286 (234045–97594)	163969 (223758–108875)
**Southern**	45–49	Women	≤5 g/d by 2030	5.9 (5.9–5.9)	11.5 (9.1–13.6)	39.4 (56.9–26.8)	50 (56.7–41.6)	102472 (153872–68491)	699214 (808908–577547)	41637 (63742–24566)	145921 (183948–108992)	201147 (309618–130788)	1403370 (1631403–1149585)	81443 (127931–46908)	292642 (371064–218257)

**Southern**	45–49	Men	≤5 g/d by 2030	6.2 (6.2–6.2)	12 (9.7–14)	41 (58.2–28.3)	51.8 (58.1–43.6)	175268 (247729–123930)	1182171 (1350771–1011619)	125007 (181155–87727)	219191 (267891–172063)	345951 (497734–236584)	2377296 (2730381–2012991)	246512 (366550–165407)	440305 (540100–344185)
**Southern**	50–54	Women	≤5 g/d by 2030	6 (6–6)	11.7 (9.5–13.5)	34.8 (45.5–25.8)	43.4 (49–36.9)	119258 (159870–88514)	556697 (654654–465339)	45559 (67279–29901)	117182 (150792–87426)	251163 (344113–183507)	1196141 (1412481–990162)	95759 (143961–61986)	251806 (325097–186746)
**Southern**	50–54	Men	≤5 g/d by 2030	6 (6–6)	11.6 (9.4–13.5)	34.7 (45.3–25.9)	43.3 (48.9–36.4)	210229 (280809–157833)	910523 (1054256–775596)	121361 (166813–87726)	168468 (210476–130617)	443436 (604247–325403)	1952949 (2274696–1646355)	255791 (356933–181164)	361419 (452711–279160)
**Southern**	55–59	Women	≤5 g/d by 2030	5.6 (5.6–5.6)	10.7 (8.9–12.5)	32.3 (42–24.3)	40.8 (46.5–35)	163663 (215184–127265)	969248 (1094928–837676)	97505 (128516–73149)	223443 (267682–182899)	390991 (531443–292223)	2392462 (2729374–2031038)	232929 (316983–169944)	550944 (665649–444862)
**Southern**	55–59	Men	≤5 g/d by 2030	6.1 (6.1–6.1)	11.8 (9.9–13.7)	35 (45–26.6)	43.8 (49.8–37.8)	284278 (357965–223827)	1331418 (1511156–1157623)	212757 (273064–166526)	522029 (603748–443528)	685942 (888442–521600)	3305220 (3796583–2839767)	514535 (677297–387164)	1295825 (1518348–1082032)
**Southern**	60–64	Women	≤5 g/d by 2030	5.2 (5.2–5.2)	10.1 (8.3–11.9)	25 (29.6–20.7)	31.1 (36.2–26.1)	175661 (210547–147562)	629068 (721183–543179)	136283 (162884–111931)	144104 (171018–118742)	439039 (538281–357296)	1631184 (1908807–1367926)	340271 (416597–272040)	373572 (450340–301041)
**Southern**	60–64	Men	≤5 g/d by 2030	5.6 (5.6–5.6)	10.8 (8.9–12.5)	26.4 (31.1–22)	32.7 (38.1–27.5)	273541 (319036–231006)	844640 (968764–728515)	243936 (288065–204351)	330347 (389919–282925)	689327 (822413–568846)	2207447 (2584999–1859043)	614967 (740385–499786)	863121 (1038990–722739)
**Southern**	65–69	Women	≤5 g/d by 2030	4.6 (4.6–4.6)	8.9 (7.4–10.4)	22.4 (26.7–18.2)	27.8 (32.6–23.1)	249691 (290413–214500)	926103 (1046424–811880)	208469 (246648–178451)	482915 (555786–420801)	539332 (648768–445909)	2083723 (2413727–1768678)	449816 (550801–372699)	1087866 (1277785–912514)
**Southern**	65–69	Men	≤5 g/d by 2030	5.3 (5.3–5.3)	10.2 (8.6–12)	25.4 (30.2–20.8)	31.3 (36.6–26.3)	371493 (432215–319606)	1282968 (1450119–1122412)	334511 (388974–286640)	816144 (930517–706982)	820958 (977515–684530)	2939691 (3419670–2499357)	739398 (882141–613142)	1877397 (2186188–1580291)
**Southern**	70–79	Women	≤5 g/d by 2030	6.6 (4.7–8)	13.2 (8.8–17.1)	40.1 (66.2–22)	51.7 (69.8–27.4)	1743877 (2232060–1366749)	6032862 (6944726–5165817)	1248333 (1592437–982342)	1853602 (2220086–1519866)	3977196 (5178760–3032851)	13978658 (16237622–11794612)	2877909 (3725285–2203522)	4362313 (5268633–3527816)
**Southern**	70–79	Men	≤5 g/d by 2030	5.9 (4.6–6.2)	11.1 (8.3–13.7)	35.3 (58.8–21.3)	46 (61.7–26.7)	2429074 (3152759–1891918)	10375642 (11967972–8840421)	1740870 (2255723–1347876)	3439311 (4128990–2825291)	5436767 (7194625–4124184)	23735760 (27726437–19889874)	3932504 (5191830–2964793)	7977403 (9682935–6439409)

Tan M, *et al. bmjnph* 2022; 5:e000408. doi: 10.1136/bmjnph-2021-000408

References for supplementary materials

1. National Bureau of Statistics of China. China Statistical Yearbook - 2018, 2019.
2. Census and Statistics Department. Population Estimates 2019 [Available from: https://www.censtatd.gov.hk/hkstat/sub/sp150.jsp?tableID=002&ID=0&productType=8 accessed 10.01.2019.
3. Tan M, He FJ, Wang C, et al. Twenty-Four-Hour Urinary Sodium and Potassium Excretion in China: A Systematic Review and Meta-Analysis. *J Am Heart Assoc* 2019;8:e012923.
4. Zhang J, Liu X, Tan Z, et al. Mapping of the north-south demarcation zone in China based on GIS. *J Lanzhou Univ Nat Sci* 2012;48:28-33.
5. Li Y, Zhang P, Wu J, et al. Twenty-Four-Hour Urinary Sodium and Potassium Excretion and Their Associations With Blood Pressure Among Adults in China: Baseline Survey of Action on Salt China. *Hypertension* 2020;76:1580-88.
6. Zhang P, He FJ, Li Y, et al. Reducing Salt Intake in China with "Action on Salt China" (ASC): Protocol for Randomized Controlled Trials. *JMIR Res Protoc* 2020;9:e15933.
7. McLean RM. Measuring population sodium intake: a review of methods. *Nutrients* 2014;6:4651-62.
8. He FJ, Zhang P, Luo R, et al. An Application-based programme to reinforce and maintain lower salt intake (AppSalt) in schoolchildren and their families in China. *BMJ Open* 2019;9:e027793.
9. Zhao D, Liu J, Wang M, et al. Epidemiology of cardiovascular disease in China: current features and implications. *Nat Rev Cardiol* 2019;16:203-12.
10. Zhang G, Yu C, Zhou M, et al. Burden of Ischaemic heart disease and attributable risk factors in China from 1990 to 2015: findings from the global burden of disease 2015 study. *BMC Cardiovasc Disord* 2018;18:18.
11. Wang W, Jiang B, Sun H, et al. Prevalence, Incidence, and Mortality of Stroke in China: Results from a Nationwide Population-Based Survey of 480 687 Adults. *Circulation* 2017;135:759-71.
12. Gu D, Kelly TN, Wu X, et al. Blood pressure and risk of cardiovascular disease in Chinese men and women. *Am J Hypertens* 2008;21:265-72.
13. Sacks FM, Svetkey LP, Vollmer WM, et al. Effects on blood pressure of reduced dietary sodium and the Dietary Approaches to Stop Hypertension (DASH) diet. DASH-Sodium Collaborative Research Group. *N Engl J Med* 2001;344:3-10.
14. MacGregor GA, Markandu ND, Sagnella GA, et al. Double-blind study of three sodium intakes and long-term effects of sodium restriction in essential hypertension. *Lancet (London, England)* 1989;2:1244-47.
15. He FJ, Ma Y, Campbell NRC, et al. Formulas to Estimate Dietary Sodium Intake From Spot Urine Alter Sodium-Mortality Relationship. *Hypertension* 2019;74:572-80.
16. He FJ, Tan M, Ma Y, et al. Salt Reduction to Prevent Hypertension and Cardiovascular Disease: JACC State-of-the-Art Review. *J Am Coll Cardiol* 2020;75:632-47.
17. Hendriksen MAH, Geleijnse JM, van Raaij JMA, et al. Identification of differences in health impact modelling of salt reduction. *PLoS One* 2017;12:e0186760.
18. Lewington S, Clarke R, Qizilbash N, et al. Age-specific relevance of usual blood pressure to vascular mortality: a meta-analysis of individual data for one million adults in 61 prospective studies. *Lancet* 2002;360:1903-13.
19. Staessen JA, Wang JG, Thijs L. Cardiovascular prevention and blood pressure reduction: a quantitative overview updated until 1 March 2003. *J Hypertens* 2003;21:1055-76.

20. Staessen JA, Wang JG, Thijs L. Cardiovascular protection and blood pressure reduction: a meta-analysis. *Lancet* 2001;358:1305-15.
21. Turnbull F, Blood Pressure Lowering Treatment Trialists C. Effects of different blood-pressure-lowering regimens on major cardiovascular events: results of prospectively-designed overviews of randomised trials. *Lancet* 2003;362:1527-35.
22. Adler AI, Stratton IM, Neil HA, et al. Association of systolic blood pressure with macrovascular and microvascular complications of type 2 diabetes (UKPDS 36): prospective observational study. *BMJ* 2000;321:412-9.
23. He FJ, Li J, MacGregor GA. Effect of longer term modest salt reduction on blood pressure: Cochrane systematic review and meta-analysis of randomised trials. *BMJ* 2013;346:f1325.
24. Huang L, Trieu K, Yoshimura S, et al. Effect of dose and duration of reduction in dietary sodium on blood pressure levels: systematic review and meta-analysis of randomised trials. *BMJ* 2020;368:m315.
25. Juraschek SP, Woodward M, Sacks FM, et al. Time Course of Change in Blood Pressure From Sodium Reduction and the DASH Diet. *Hypertension* 2017;70:923-29.
26. Forte JG, Miguel JM, Miguel MJ, et al. Salt and blood pressure: a community trial. *J Hum Hypertens* 1989;3:179-84.
27. He FJ, Pombo-Rodrigues S, Macgregor GA. Salt reduction in England from 2003 to 2011: its relationship to blood pressure, stroke and ischaemic heart disease mortality. *BMJ Open* 2014;4:e004549.
28. Laatikainen T, Pietinen P, Valsta L, et al. Sodium in the Finnish diet: 20-year trends in urinary sodium excretion among the adult population. *Eur J Clin Nutr* 2006;60:965-70.
29. Karppanen H, Mervaala E. Sodium intake and hypertension. *Progress in cardiovascular diseases* 2006;49:59-75.
30. Wang M, Moran AE, Liu J, et al. A Meta-Analysis of Effect of Dietary Salt Restriction on Blood Pressure in Chinese Adults. *Glob Heart* 2015;10:291-99 e6.
31. Ettehad D, Emdin CA, Kiran A, et al. Blood pressure lowering for prevention of cardiovascular disease and death: a systematic review and meta-analysis. *Lancet* 2016;387:957-67.
32. Law MR, Morris JK, Wald NJ. Use of blood pressure lowering drugs in the prevention of cardiovascular disease: meta-analysis of 147 randomised trials in the context of expectations from prospective epidemiological studies. *BMJ* 2009;338:b1665.
33. Lawes CM, Rodgers A, Bennett DA, et al. Blood pressure and cardiovascular disease in the Asia Pacific region. *J Hypertens* 2003;21:707-16.
34. Singh GM, Danaei G, Farzadfar F, et al. The age-specific quantitative effects of metabolic risk factors on cardiovascular diseases and diabetes: a pooled analysis. *PLoS One* 2013;8:e65174.
35. Institute for Health Metrics and Evaluation. GBD Results Tool 2020 [Available from: http://ghdx.healthdata.org/gbd-results-tool accessed 14.10.2019.
36. Kypridemos C, Guzman-Castillo M, Hyseni L, et al. Estimated reductions in cardiovascular and gastric cancer disease burden through salt policies in England: an IMPACTNCD microsimulation study. *BMJ Open* 2017;7:e013791.
37. Weinstein MC, Coxson PG, Williams LW, et al. Forecasting coronary heart disease incidence, mortality, and cost: the Coronary Heart Disease Policy Model. *Am J Public Health* 1987;77:1417-26.
38. Asaria P, Chisholm D, Mathers C, et al. Chronic disease prevention: health effects and financial costs of strategies to reduce salt intake and control tobacco use. *The Lancet* 2007;370:2044-53.

39. Boshuizen HC, Lhachimi SK, van Baal PH, et al. The DYNAMO-HIA model: an efficient implementation of a risk factor/chronic disease Markov model for use in Health Impact Assessment (HIA). *Demography* 2012;49:1259-83.
40. Hendriksen MAH, Hoogenveen RT, Hoekstra J, et al. Potential effect of salt reduction in processed foods on health1–3. *The American Journal of Clinical Nutrition* 2014;99:446-53.
41. Scarborough P, Nnoaham KE, Clarke D, et al. Modelling the impact of a healthy diet on cardiovascular disease and cancer mortality. *Journal of Epidemiology and Community Health* 2012;66:420-26.
42. Mozaffarian D, Fahimi S, Singh GM, et al. Global Sodium Consumption and Death from Cardiovascular Causes. *New England Journal of Medicine* 2014;371:624-34.
43. Bibbins-Domingo K, Chertow GM, Coxson PG, et al. Projected effect of dietary salt reductions on future cardiovascular disease. *New England Journal of Medicine* 2010;362:590-99.
44. Cobiac LJ, Vos T, Veerman JL. Cost-effectiveness of interventions to reduce dietary salt intake. *Heart* 2010;96:1920-5.

Tan M, *et al. bmjnph* 2022; 5:e000408. doi: 10.1136/bmjnph-2021-000408
